# Endophytic fungi from the roots of horseradish (*Armoracia rusticana*) and their interactions with the defensive metabolites of the glucosinolate - myrosinase - isothiocyanate system

**DOI:** 10.1186/s12870-018-1295-4

**Published:** 2018-05-09

**Authors:** Zsolt Szűcs, Tamás Plaszkó, Zoltán Cziáky, Attila Kiss-Szikszai, Tamás Emri, Regina Bertóti, László Tamás Sinka, Gábor Vasas, Sándor Gonda

**Affiliations:** 1 Department of Botany, Division of Pharmacognosy, University of Debrecen, Egyetem tér 1, Debrecen, H-4010 Hungary; 2 Department of Organic Chemistry, University of Debrecen, Egyetem tér 1, Debrecen, H-4010 Hungary; 3 Department of Biotechnology and Microbiology, University of Debrecen, Egyetem tér 1, Debrecen, H-4010 Hungary; 4 Department of Pharmacognosy, Semmelweis University, Üllői út 26, Budapest, H-1085 Hungary; 5 Agricultural and Molecular Research and Service Institute, University of Nyíregyháza, Sóstói str. 31/b, Nyíregyháza, H-4400 Hungary

**Keywords:** Myrosinase, Endophytes, Glucosinolate, Isothiocyanate, Fungal enzyme

## Abstract

**Background:**

The health of plants is heavily influenced by the intensively researched plant microbiome. The microbiome has to cope with the plant’s defensive secondary metabolites to survive and develop, but studies that describe this interaction are rare. In the current study, we describe interactions of endophytic fungi with a widely researched chemical defense system, the glucosinolate - myrosinase - isothiocyanate system. The antifungal isothiocyanates are also of special interest because of their beneficial effects on human consumers.

**Results:**

Seven endophytic fungi were isolated from horseradish roots (*Armoracia rusticana*), from the genera *Fusarium*, *Macrophomina*, *Setophoma*, *Paraphoma* and *Oidiodendron.* LC-ESI-MS analysis of the horseradish extract incubated with these fungi showed that six of seven strains could decompose different classes of glucosinolates. Aliphatic, aromatic, thiomethylalkyl and indolic glucosinolates were decomposed by different strains at different rates. SPME-GC-MS measurements showed that two strains released significant amounts of allyl isothiocyanate into the surrounding air, but allyl nitrile was not detected. The LC-ESI-MS analysis of many strains’ media showed the presence of allyl isothiocyanate - glutathione conjugate during the decomposition of sinigrin. Four endophytic strains also accepted sinigrin as the sole carbon source. Isothiocyanates inhibited the growth of fungi at various concentrations, phenylethyl isothiocyanate was more potent than allyl isothiocyanate (mean IC_50_ was 2.30-fold lower).

As a control group, ten soil fungi from the same soil were used. They decomposed glucosinolates with lower overall efficiency: six of ten strains had insignificant or weak activities and only three could use sinigrin as a carbon source. The soil fungi also showed lower AITC tolerance in the growth inhibition assay: the median IC_50_ values were 0.1925 mM for endophytes and 0.0899 mM for soil fungi.

**Conclusions:**

The host’s glucosinolates can be used by the tested endophytic fungi as nutrients or to gain competitive advantage over less tolerant species. These activities were much less apparent among the soil fungi. This suggests that the endophytes show adaptation to the host plant’s secondary metabolites and that host metabolite specific activities are enriched in the root microbiome. The results present background mechanisms enabling an understanding of how plants shape their microbiome.

**Electronic supplementary material:**

The online version of this article (10.1186/s12870-018-1295-4) contains supplementary material, which is available to authorized users.

## Background

It is now well established that the plant microbiome affects the health of plants, just as the much more researched human microbiome influences ours [1]. Of course, the complex groups of microbes also interact with the plant metabolites. Some natural products are co-products of the endophytes and the plants; several endophytes can selectively transform the plant’s secondary metabolites [2]. Endophytic fungi are an intensively studied subset of the plant microbiome. A huge amount of scientific literature is gathering on this group, which is not that straightforward to define [3, 4]. Various definitions exist, but they are usually considered non-pathogenic strains that live inside plants without causing apparent symptoms [3, 4]. Endophytes are very good subjects for drug discovery. The possibility of finding new natural products is a significant driving force of the research of these fungi [5]. Endophytes are also well studied for production of different enzymes that operate under special conditions, for example, endophytes isolated from plants living in saline habitats, harbor enzymes that can operate at high salt concentrations [6]. Their beneficial interaction with plants also has potential uses in crop protection and plant health promotion (e.g. biocontrol, salt tolerance [7, 8]). However, the non-pathogenic lifestyle can not be considered stable [3, 4]. “True” endophytes sometimes become latent pathogens, and this has severe agricultural impact [9, 10]. Despite all the above, the endophytes’ interaction with the plant defense metabolites is not that studied.

**Fig. 1:**
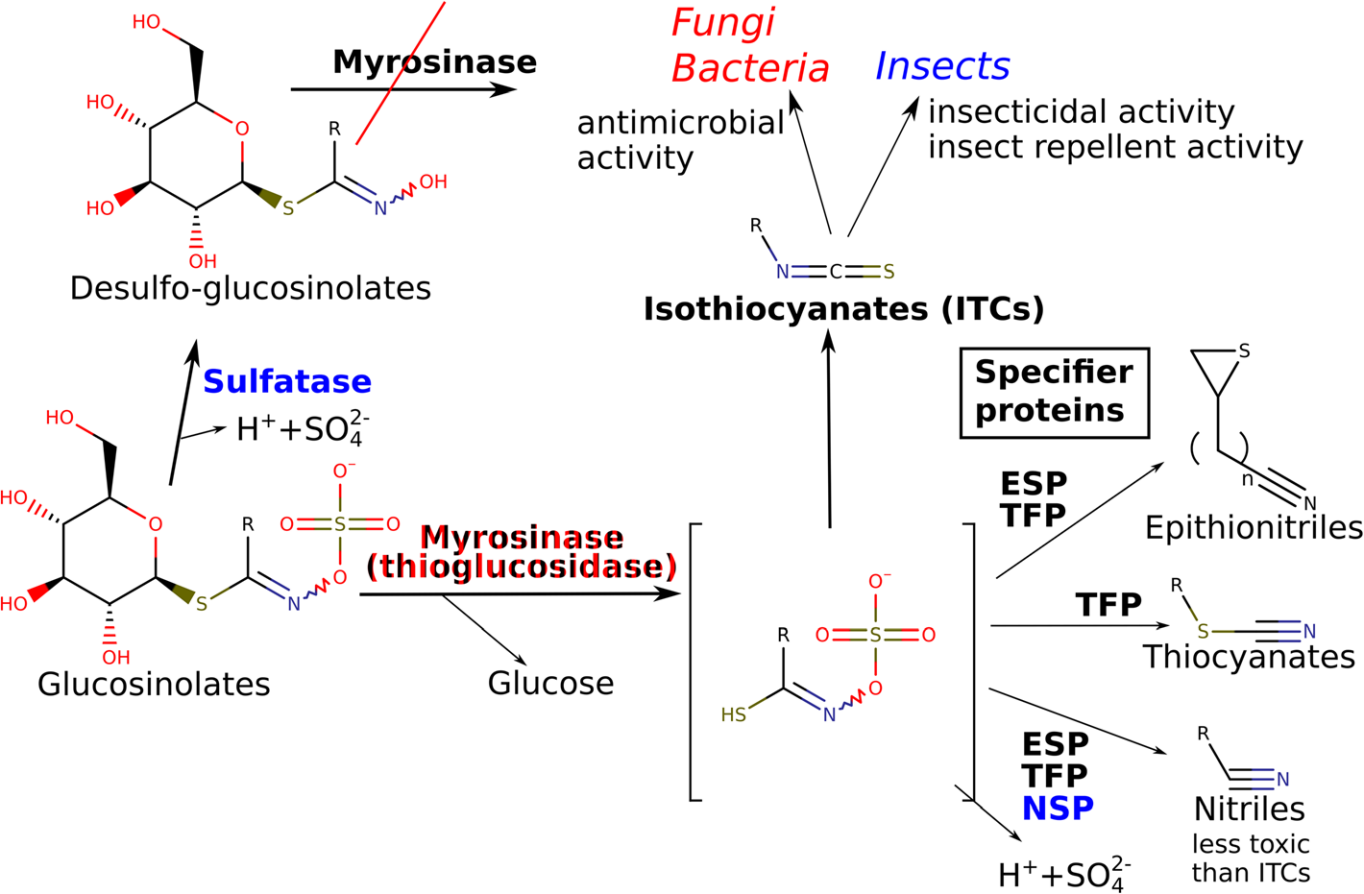
The glucosinolate – myrosinase – isothiocyanate system and its interactions with fungi and members of the ecosystem. Known sources of enzymes are color coded: Plants: black, fungi or bacteria: red, insects: blue. Abbreviations: ESP, epithionitrile specifier protein, NSP, nitrile specifier protein, TFP, thiocyanate-forming protein. References: [13, 56, 61, 62]

The glucosinolate (GSL) - myrosinase (MYR) - isothiocyanate (ITC) chemical defense system is present in Brassicaceae, Capparaceae, Resedaceae and Moringaceae. It is one of the most researched plant defense systems [11]. Myrosinase (EC 3.2.1.147, thioglucoside glucohydrolase) catalyzes the reaction between a glucosinolate and water to yield glucose and a thiohydroximate. The free thiohydroximate subsequently spontaneously rearranges to isothiocyanates and byproducts (Fig. 1) [12, 13]. Most isothiocyanates are extremely pungent and exert various bioactivities due to their reactive -NCS groups, which results in strong antimicrobial, insecticidal and other effects [12]. This chemical defense system is present in vegetables of the Brassicaceae with various glucosinolates. Interestingly, the isothiocyanates have beneficial effects on humans, making the Brassicaceae crops healthy functional foods. Regular consumption of low amounts of isothiocyanates can prevent different human diseases [12].

Glucosinolates are chemically stable and are separated from their activating enzyme – MYR – by compartmentalization. They typically come in contact *in planta* when the plant tissue is damaged. There is a considerable variability of the actual separation strategy that depends on species, organ, developmental stage and many other factors [11]. Myrosinase and the GSLs are usually stored in separate cells, but this is not always the case. The GSL - ITC system is therefore considered a “chemical bomb” that is triggered by the attack of pathogens and herbivores [11]. The isothiocyanates were shown to have potent antifungal activity in many papers (e.g. [14–16]). It is an interesting question how endophytes tolerate the isothiocyanates of the plants. Actually, myrosinase activity can benefit microorganisms, as it enables them to obtain glucose from the thioglucoside. For endophytes, there would be an additional benefit as it would give them the option to disarm the “chemical bomb” of the host. Of course, they have to cope with the toxicity of the released ITCs or prevent its release (as shown for an *Aspergillus* sp. strain [17]). MYR activity was shown to be present in many microbial organisms including *Aspergillus sp.*, *Fusarium sp.* [18], *Citrobacter* [19], *Enterobacter* [20] as well as many other species. Strains capable of metabolizing glucosinolates are part of the human gut microbial community [21–23].

Regarding effects of endophytes on the host’s secondary metabolome, not many studies have been published to date. A study has shown that the endophytes specifically modify the plant metabolome to their own benefit. In particular, deglycosylation of flavonoid glycosides by the fungus *Paraconiothyrium variabile* was observed. The deglycosylated flavonoids displayed significant beneficial effects on the hyphal growth of germinated spores [24]. In addition, cases of selective *in vitro* modifications of plant secondary metabolites have been reported [2].

The effects of plant metabolites on endophytic fungi are examined in more detail. Studies suggest that chemical constituents actually drive the development of the microbiome around the plant roots. Microbes that can tolerate these metabolites are enriched in the rhizosphere. For example, *Fusarium* and *Rhizopus* species that can decompose the glucosinolate exudate were abundant in the soil around roots of Brassicaceae plants [25]. Some plant pathogens (e.g. *Phoma lingam* and *Verticillium dahliae*) can also decompose glucosinolates [26]. It is suggested that the decomposition of sinigrin is a detoxification process, as sinigrin hydrolysis had no effects on growth or spore germination of the tested fungi. Therefore, it was argued that the pathogens can perhaps use this ability to overcome the plant defenses. The pathogen *Alternaria brassicicola* adapts to the antifungal allyl isothiocyanate (AITC) via increased transcription of oxidative stress response genes. These include glutathione S-transferases, thioredoxins, glutathione peroxidases, γ-glutamylcysteine synthetases [27].

Though there are many reports about fungi with myrosinase activity, the community of endophytic fungi from Brassicaceae plants were not tested for myrosinase. As the GSL - MYR - ITC system is usually considered a chemical defense system against fungi, the presence of this activity would make this system more complex. Also, in such an approach, a direct comparison with some control group of fungi would be desirable so that the specificity of the phenomenon can be assessed.

The aim of the current study was to test the hypothesis that endophytes interfere with the GSL - MYR - ITC chemical defense system of plants. The main questions are: 1., Do endophytes tolerate the ITCs of the host better than non-endophytic fungi? 2., Can endophytes decompose GSLs? Can they use them as a carbon source? 3., How specific is the activity of the endophytes? To answer the above questions, we used isolated fungal root endophytes from *Armoracia rusticana* (horseradish), a Brassicaceae crop with a very high abundance of ITCs. The strains were grown on horseradish extract to test their glucosinolate decomposing capacity and their ability to produce various decomposition products. Their ITC tolerance and ability to use GSLs as a sole carbon source was also examined. As a reference group, a set of soil fungi from the same soil was used.

## Methods

### Chemicals

Reagents were of analytical purity. Media components (peptone, glucose monohydrate, sodium nitrate, potassium hydrogenphosphate, magnesium sulfate, potassium chloride, ferrous sulfate) were from Reanal (Budapest, Hungary). Allyl isothiocyanate, allyl cyanide, 2-phenylethyl isothiocyanate and phenylpropionitrile and glutathione (reduced form) were from Sigma Aldrich (MO, USA). Pure glucosinolates were from Phytoplan (Germany). LC-MS grade acetonitrile, water and formic acid were purchased from Fisher Scientific (Belgium). Solvents (1-propanol, 2-propanol) and media additives (streptomycin, chloramphenicol and dichloran) were from VWR. Double distilled water was used throughout the study.

### Standard microbiological media

The following media and compositions were used: Saboraud Glucose agar broth or agar (SGB or SGA, 40 g / L glucose monohydrate, peptone 10 g / L, pH 5.6 ± 0.2); potato dextrose agar (PDA, potato infusion equivalent to 200 g / L, 20 g / L glucose monohydrate, pH 5.6 ± 0.2); Czapek-Dox medium (CzD, 3.0 g; NaNO_3_; 1.0 g K_2_HPO_4_; 0.5 g MgSO_4_·7H_2_O; 0.5 g KCl and 0.01 g FeSO_4_·7H_2_O / L, pH 7.3 ± 0.2). Media were solidified with 2% agar, where appropriate.

### Isolation of endophytes

Endophytic fungi from horseradish (*Armoracia rusticana*) were used for the study. Healthy horseradish roots from the cultivated populations, from the region of Újléta or Debrecen were used. Collection of the roots complied with local legislation and guidelines. Horseradish roots were surface-sterilized with 4-fold diluted commercial bleaching solution, followed by several rinses with sterile distilled water. Afterwards, the roots were cut into pieces (approximately 2 × 2 × 1 cm in size) and placed on SGA for endophyte isolation. Surface sterility was checked by imprinting the surface-sterilized plant tissue onto SGA plates, and/or spreading aliquots of the last rinse fluid on the same type of medium. When no growth in these negative control plates was observed, the explant batch was considered free of surface contaminants, as proposed by [28]. The plates were sealed and stored at room temperature. The fungi appeared at the edge of the explants were considered endophytes. The isolated fungi are referred to as *E1*-*E7* throughout the manuscript.

### Isolation of soil fungi

For comparison with the endophytes, a set of soil fungi were obtained from the Debrecen site, where some of the horseradish roots were from. Sampling was performed in June of 2016. No plants were present at the sampling point. About 1 g of soil and 10 mL of sterile water were mixed vigorously to form a suspension. After 10^3^-fold dilution of the above stock suspension, 100 μL of the diluted soil suspension was spread onto PDA or SGA plates containing 50 mg L^− 1^ streptomycin and 50 mg L^− 1^ chloramphenicol. A subset of isolation plates also contained 2 mg L^− 1^ dichlorane. The appeared fungi were subcultured to obtain pure strains and used for the later study. Of these soil fungi, 10 apparently different strains were randomly selected for the current work. These are referred to as *S1*-*S10* throughout the manuscript.

### Identification of strains

Pure strains were grown in shaken cultures, lyophilized, and their DNA was purified for identification of species. DNA sequencing was carried out as described earlier [29] using the following primer pairs: ITS1, ITS4, (ITS); Bt2a, Bt2b (β-tubulin); act-512F, ACT-783R (α-actin) and EF1-728F, EF1-986R (translation elongation factor 1α). The obtained sequences were deposited at the GenBank nucleotide sequence database under the following accession numbers: KP191628-KP191640. Isolates were identified by comparison of their sequences with sequences available at GenBank (http://www.ncbi.nlm.nih.gov/genbank), Q-bank Fungi (http://www.q-bank.eu/fungi/) and Fusarium ID (http://isolate.fusariumdb.org) databases.

### Preparation of horseradish root extract (HRE)

To examine the fungal decomposition ability against different glucosinolates, a growth medium from the host plant was prepared and used for the study. This aqueous liquid contained the metabolome of the horseradish at approximately original concentration. About 500–1000 g of healthy horseradish roots were cut into large pieces and cooked in water for 30 min to inactivate the myrosinase. Thereafter, the cooked roots were homogenized with MeOH in a 3:2 solvent to fresh weight ratio and subsequently boiled (80 °C) under reflux for 30 min. The extract was filtered and evaporated to dryness in a rotary evaporator. Then, the dry residue was dissolved in water corresponding to the original water content of the roots (70% water content is typical). The liquid was filtered sterile on 0.20 μm PES membranes after a series of prefiltrations, and was stored at − 24 °C before use. If necessary, the liquid was supplemented with 2% autoclaved agar.

### Preparation of fungal inoculums

For the initiation of experiments, liquid suspensions of the fungi were used to ensure uniform inoculation of wells and samples. Fungi were grown in 30 mL of either 2% malt extract broth, Czapek Dox medium or HRE in shaken cultures at room temperature, 200 rpm. The time necessary for full growth was usually 3–9 days. Sterile glass homogenizers were applied to disintegrate the washed fungal mycelia without the use of quartz sand. The suspensions of the fungal inoculums were stored at 4 °C before the experiments. As the species differed in morphology and the ability to form conidia, dry weight per mL was chosen as a means of standardization. Dry weight was determined gravimetrically after lyophilization of the inoculum suspensions.

### Incubation of horseradish extract with fungi

The fungi were incubated in the previously described horseradish extract to detect their effects on the horseradish secondary metabolites. Five mL aliquots of HRE were inoculated with fungal inoculums equivalent to 40 μg dry weight per mL. Thereafter, to correct for dilution, the liquid was separated into test tubes so that each tube contained an amount equivalent to 250 μL HRE. The tubes were placed in a sterile plastic container where sterile bidistilled water provided high relative humidity to minimize evaporation during the study. Samples were frozen and stored at − 24 °C until further processing. At least 3 replicates were collected for each data point. Control was not inoculated. The experiment was run for 16 days. Sampling times were based on preliminary experiments. To provide samples at the different points of the growth curves, the seven endophytic strains were sampled at different days. A similar experiment was run with the soil fungi, in this case, only the 16-day samples were processed. After lyophilization of the samples, the samples were dissolved in 250 μL 10% 1-PrOH to help centrifugation and reduce the dissolution of viscous polysaccharides. The fungal pellet was liophilized and the dry mass was measured to obtain the growth curve of the different strains.

### Measurement of glucosinolate decomposition (LC-ESI-MS)

The glucosinolate decomposition by endophytic and soil fungi was quantified / screened in LC-ESI-MS. The UHPLC system (Dionex Ultimate 3000RS) was coupled to a Thermo Q Exactive Orbitrap mass spectrometer (Thermo Fisher Scientific Inc., Waltham, USA) equipped with an electrospray ionization source (ESI). The column was a Phenomenex Kinetex XB-C_18_ column (100 mm × 2.1 mm × 2.6 μm). Oven temperature was maintained at 30 °C; the flow rate was 250 μL min^− 1^. Eluent A was water and eluent B was acetonitrile, both contained 0.1% formic acid. The following gradient elution program was used: 0 min, 5% B, 0–2 min, 5% B; 2–5 min, 25% B; 5–6 min, 60% B; 6–7 min, 100% B; 7–9 min, 100% B; 9–10 min, 5% B, 10–18 min, 5% B. One μL of the 200-fold dilutions (with MeOH) of the redissolved HRE samples was injected in every run. With a serial dilution, it was shown that this dilution is within the linear range of the analytes of interest. The Q Exactive hybrid quadrupole-orbitrap mass spectrometer operated in negative ion mode with the following parameters: capillary temperature 320 °C, spray voltage 3.8 kV, the resolution was set to 70,000. The mass range scanned was 150–1000 m/z. The maximum injection time was 100 ms. Sheath gas and aux flow rates were 32 and 7 arb, respectively. The raw data were processed by the XCMS Online platform [30], using parameters suggested for the Orbitrap instrument, with minor modifications. The peak lists were further processed in R [31], using ggplot2 [32]. For quantification of major GSLs, five-point calibration curves were constructed using sinigrin and gluconasturtiin. The calibration curve spanned the concentration range of 0.01–5 μg mL^− 1^. For the quantification of GSH, a calibration curve in the range of 0.01–5 μg mL^− 1^ was used. For GSH-AITC quantification, the derivatization reaction was adapted from our recent study [33]. A five point calibration curve was constructed by reacting 0.001–1 μg mL^− 1^ AITC with excess GSH at pH 7.5, in NH_4_OAc buffer [33].

### SPME-GC-MS of microbial volatile organic constituents

Typical voltatile glucosinolate decomposition products (isothiocyanates and nitriles) were sought by a newly developed SPME-GC-MS method. Ten mL of horseradish extract with 2% agar was poured in Petri dishes 9 cm in diameter. This amount contains approximately 60 μmol sinigrin. The plate was inoculated with 100 μL aliquots of fungal inoculums that were spread on the surface of the medium. The cultures were grown upside down. Five mg of activated charcoal was placed in sterile tube caps in the lid of the Petri dishes to adsorb the volatile constituents from the air above the fungal culture. The sorbents were replaced daily. The charcoal samples were stored at − 24 °C before analysis. Elution from the charcoal was accomplished by methyl acetate and the resulting liquid was directly injected into GC-MS. Some additional technical details and the steps of this optimization are given in the Additional file. Measurement parameters were as follows: split ratio was 10:1; inlet temperature was 150 °C. Time program: Initial oven temperature was 35 °C, held for 3 min, followed by a 15 °C/min temperature gradient to 90 °C, then 50 °C/min to 320 °C and held for 2 min. A HP-5MS 5% Phenyl Methyl Siloxane column was used. Carrier gas was He; flow rate was 1 mL min^− 1^. Solvent delay was 2.75 min.

As five-point calibration curves, serial dilutions of authentic standards of allyl cyanide, allyl isothiocyanate, phenylpropionitrile and 2-phenylethyl isothiocyanate were used.

### Glucosinolate sinigrin as a sole carbon source for fungal growth

In this experiment, the growth of the fungal strains on a synthetic medium was tested. A plant defensive metabolite, sinigrin was added as the sole carbon source. The inoculum volume was minimized to limit the amount of nutrient carryover. The growth medium was Czapek Dox medium with sinigrin, added at a concentration equimolar to 2% sucrose. As a positive control, the same medium with 2% glucose was used. Water served as negative control. The experiment was run in 96-well plates for 12 days. Hundred μl of medium were added to each well. All media were sterile filtered using PES filters. The fungal growth was tested by measuring the increase in absorbance by a plate reader at 800 nm. The changes were confirmed by visual investigation. Growth was tested in four replicates.

### Determination of IC_50_ values for growth inhibition of fungi by allyl isothiocyanate and 2-phenylethyl isothiocyanate

The growth inhibitory effect of the plant defensive compounds were investigated on the fungi. The endophytes and the soil fungi were directly compared. Aliquots of Saboraud Glucose Broth (SGB) media (500 μL) were inoculated with fungi in 24-well plates. Growth was monitored by a plate reader at 800 nm, using 5 × 5 points of absorbance determination evenly distributed within the well, thereby allowing quantification of growth of filamentous fungi. When growth started and an increase in absorbance was found, the cultures were treated with 5 μL aliqouts of diluted AITC or PEITC. Serial dilution was performed with DMSO. As a preliminary study, final concentrations of both AITC and PEITC were 0.25, 2.5, 25, 250 μg mL^− 1^. A 2500 μg mL^− 1^ equivalent treatment was also tested. In this case, the final concentration is not accurate as the medium becomes saturated with AITC or PEITC. A later experiment added several other concentrations if resolution was insufficient to determine an IC_50_ (0.445, 0.791, 1.406, 4.45, 7.91, 14.1, 44.5, 79.1, 141, 445, 791, 1406 μg mL^− 1^ equivalent). Control was treated with DMSO. As the ITCs are volatile to some extent, a single plate was treated with a single concentration of an agent. Different fungi were therefore allowed to grow 12, 24 or 36 h before treating the whole plate at the same time. The treated plates were incubated in darkness, at room temperature for 48 h. Growth was calculated by subtracting the zero time absorbance (measured just before treatment) from the absorbance measured at 48 h after treatment. Each point subjected to an IC_50_ calculation in R (using ‘drc’ [34]) was a mean of at least 3 replicates.

### Statistical analyses

To determine whether a concentration of a metabolite significantly changes as a result of treatments, ANOVAs were run on the features independently using XCMS online [30]. In XCMS Online, each point of observation was subjected to ANOVA as a separate treatment. A point of observation consisted of the samples of a treatment at a given time, *n* ≥ 3. As many statistical hypotheses were tested, Bonferroni correction was used for these data; features were significant at *p* < 3.03e-6. After successful ANOVAs, Dunett’s test was used as a post-hoc test for comparison of multiple treatments to the controls. Either end-point data or data points with nearest control treatment points were compared (both *n* = 3). All calculations were performed in R [31].

## Results and discussion

### Endophytic fungi

Seven endophytic fungi were isolated from surface-sterilized root segments of *Armoracia rusticana.* Identification showed that the isolated endophytes belong to different taxonomic groups. The isolated strains were purified and subjected to identification via sequencing taxonomically relevant segments of their DNA (ITS, β-tubulin, α-actin and translation elongation factor 1α). By comparing the sequences with sequences available at GenBank, the isolates were found to belong to the species shown in Table 1. The set contains members of three taxonomic-functional groups: The two *Fusarium oxysporum* isolates (*E1*, *E3*) belong to the Hypocerales. This species was shown to have endophytic strains in a relative plant species, *Brassica napus* [35]. The members of the other group belong to the related clades *Pleosporales (E4–6)* and *Botryosphaeriales (E2)* [36]*. These species have also been described as endophytes* [37, 38]*. The finding of Oidiodendron cerealis (E7) is somewhat unusual, as it is a typical Ericaceous (“ericoid”) endophyte* [39], which favors hosts from the Ericaceae plant family [40].

**Table 1:** List of endophytes identified in horseradish roots

ID	Species	Sequenced gene(s)	GenBank accession numbers
*E1*	*Fusarium oxysporum* species complex	ITS, eF1	KP191628, KP191635
*E2*	*Macrophomina phaseolina*	ITS, β-tubulin	KP191629, KP191637
*E3*	*Fusarium oxysporum* species complex	ITS, eF1	KP191630, KP191636
*E4*	*Setophoma terrestris*	ITS, α-actin	KP191631, KP191638
*E5*	*Paraphoma radicina*	ITS, α-actin	KP191632, KP191639
*E6*	*Paraphoma radicina*	ITS, α-actin	KP191633, KP191640
*E7*	*Oidiodendron cerealis*	ITS	KP191634

### Glucosinolate decomposition by horseradish endophytes and soil fungi.

Several tested fungi (*Macrophomina phaseolina, Fusarium oxysporum*, *Setophoma terrestris*, *Paraphoma radicina*) were shown to decompose various glucosinolates; the endophytes were overall more efficient decomposers than the soil fungi from the same soil. The rates of decomposition depended on the side chain of the glucosinolates to some extent, as detailed later. The main method of this approach was incubation of the fungal strains in horseradish extract (HRE) as a growth medium, and quantification of glucosinolate concentrations by LC-MS. This allowed the testing of the fungi for putative myrosinase activity, but required purified strains of endophytes and soil fungi.

Various glucosinolates could be identified from the horseradish extract. LC-MS/MS examination showed that a 250 μL aliquot (equivalent to 0.295 g horseradish FW or 0.088 g horseradish DW) contained 1.42 μmol sinigrin (2.37 mg mL^− 1^), 0.077 μmol gluconasturtiin (2-phenylethyl glucosinolate) (129 μg mL^− 1^) and several, less abundant minor GSLs (Table 2). The LC-MS dataset of the incubation was subjected to automatic feature detection by XCMS Online, followed by targeted screening for glucosinolates. If a feature had chromatographic peak shape and it yielded at least two of the GSL-specific fragments in MS/MS, it was identified as a glucosinolate. For this purpose, we used the characteristic ions 259.012 (C_6_H_11_SO_9_^−^) and 96.960 (HSO_4_^−^) in MS^2^ [41, 42]. The detected minor glucosinolates included aliphatic (gluconapin, glucocochlearin), thiomethylalkyl (glucoiberin), and indolic (glucobrassicin) types as well (Table 2, Additional file 1: Figure S1). Agneta et al. has previously reported these glucosinolates in horseradish roots [43]. Though the detection sensitivity of GSLs by LC-ESI-MS is variable to some extent, several of the detected GSLs have a response ratio between 0.73–1.72 vs sinigrin [44, 45]. Therefore, the minor GSLs are estimated to be present at quantities between 0.7–40.6 nmol (each) in a 250 μL aliquot.

**Table 2:** Identified glucosinolates found in the horseradish extract. These compounds were subjected to fungal decomposition by horseradish endophytes and soil fungi

[M − H]^−^ (m/z)	Rt	Common name	Side chain	Glucosinolate class
358.0275	1.59	Sinigrin*	2-propenyl	aliphatic
372.0434	2.69	Gluconapin	3-butenyl	aliphatic
374.0591	4.23	Glucocochlearin	1-methylpropyl	aliphatic
386.0592	6.37	Glucobrassicanapin	4-pentenyl	aliphatic
422.0261	1.28	Glucoiberin*	3-(methylsulfinyl)propyl	methylthioalkyl
478.0888	7.40	Glucoibarin	7-(methylsulfinyl)heptyl	methylthioalkyl
408.0435	7.32	Glucotropaeolin	benzyl	aromatic
422.0591	9.08	Gluconasturtiin*	phenylethyl	aromatic
447.0544	8.49	Glucobrassicin*	indol-3-ylmethyl	indole
477.0649	9.29	4-Methoxyglucobrassicin	4-methoxyindol-3-ylmethyl	indole

Glucosinolates whose identity is also proven by comparison to an authentic standard are marked with an asterisk, the rest is identified from the results of [43]

Abbreviation: *Rt* retention time (min)

**Fig. 2:**
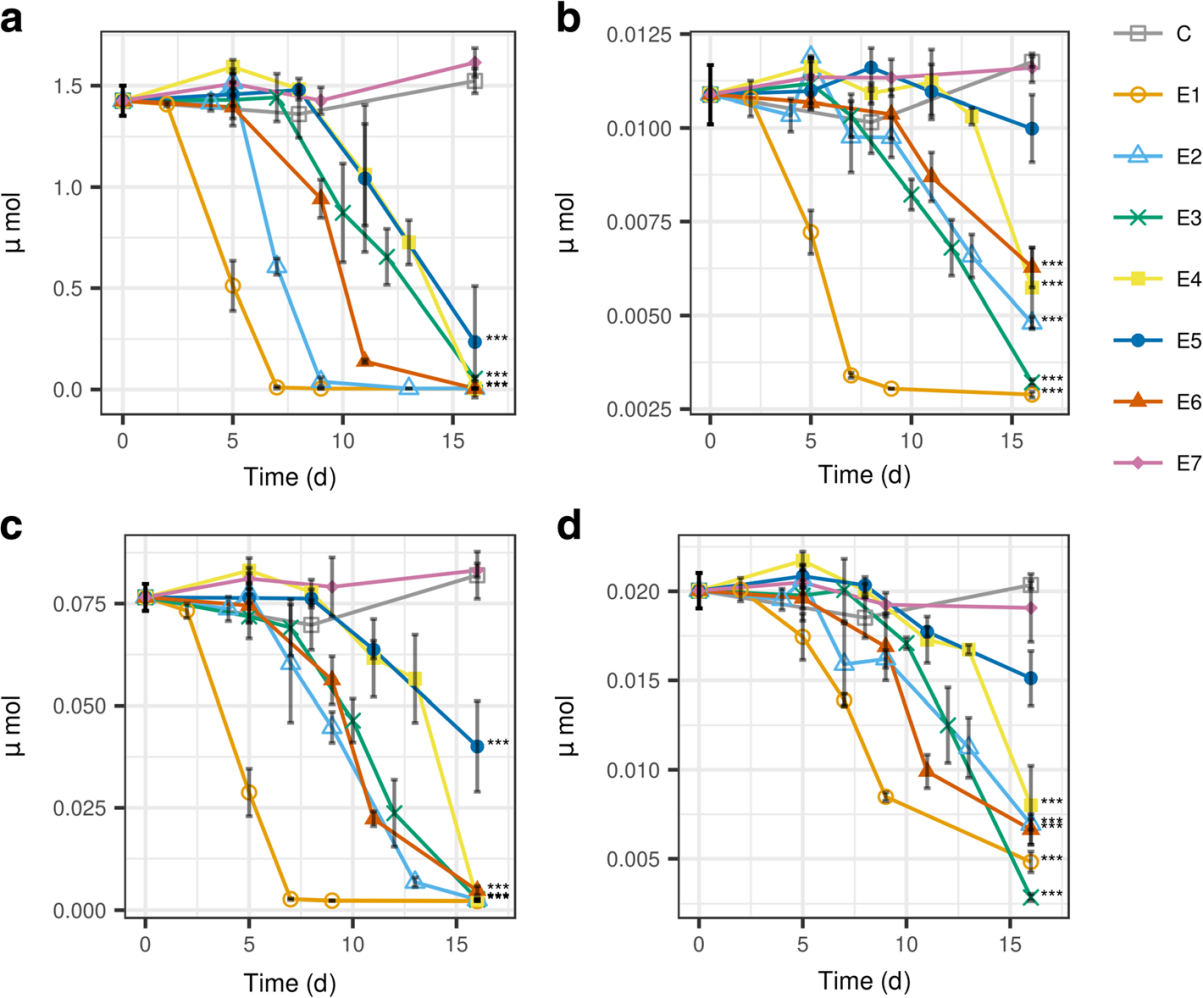
Decomposition of glucosinolates in horseradish extract inoculated by endophytic fungi from horseradish. Subplots: a., sinigrin; b., glucoiberin; c., gluconasturtiin; d., glucobrassicin. Fungi: *E1*, *Fusarium oxysporum*; *E2*, *Macrophomina phaseolina*; *E3*, *Fusarium oxysporum*; *E4*, *Setophoma terrestris*; *E*5, *Paraphoma radicina*; *E6*, *Paraphoma radicina*; *E*7, *Oidiodendron cerealis*; ***C***, control (not inoculated)*.* Statistical test: Dunnett’s test, endtime samples compared to end-time control (*n* = 3, ***, *p* < 10^− 5^; **, *p* < 10^− 4^ *, *p* < 5*10^− 4^). For the data in Table form, see Additional file 1: Table S1

In controls, the concentrations of most glucosinolates did not change significantly during the 16 day incubation period (*p* > 0.05). Six out of seven endophytic fungi successfully decomposed most or all glucosinolates (*p* > 0.05, Fig. 2, Additional file 2: Table S1). By the end of incubation, six of seven strains significantly decreased the amount of the major glucosinolate, sinigrin (*p* < 0.001, Fig. 2a). However, strain *E7* (*O. cerealis*) could not decompose any glucosinolates (Fig. 2, Additional file 1: Figure S2). The sinigrin decomposition ability was also tested for the same fungi in Saboraud glucose broth spiked with a similar amount of sinigrin. However, replacing the horseradish extract medium resulted in several false negative results for sinigrin decomposition with the same endophytic fungi (Additional file 1: Figure S3). This suggests that the presence of high amounts of GSLs in is not sufficient alone to induce enzymes that are responsible for the breakdown, despite the inherent ability to degrade glucosinolates is there (*E2*, *E4-E6*) (Fig. 2 vs Additional file 1: Figure S3). This underlines the importance of working in HRE to show the decomposition ability of the fungi. Despite the composition of HRE is an average of the different types of cells in the horseradish root, it resembles the in planta conditions better than a standard microbiological medium. Using whole plant extracts was also shown to be superior to endophyte studies by an other group in terms of the number of endophyte emergents as well as the growth character of the endophye cultures [46]. When non-myrosin cells are intracellularly penetrated by endophytes, the fungal myrosinase enzymes can decompose the glucosinolates therein. These enzymes can either be extracellular or intracellular [47].

After calculating the slope of concentration decrease for SGN and GLN, decomposition rates span the range 0.606–1.476 mM d-1 and 0.018–0.057 mM d^− 1^ respectively (Table 3). Within-species differences are evident for strains *E1* - *E3* (*F. oxysporum*) and *E5* - *E6* (*P. radicina*). While *E1* rapidly decomposed most glucosinolates already during its growth stage (1.125 mM d^− 1^ SGN; 0.057 mM d^− 1^ GLN), *E3* performed a much slower degradation only after its growth has stopped (0.606 mM d^− 1^ SGN; 0.030 mM d^− 1^ GLN) (Additional file 1: Figure S4). The same strains differed much less in the case of indolic glucosinolates (Fig. 2d, Additional file 1: Figure S2i-j). The differences between strains *E5* and *E6* were less pronounced when compared to the differences between strains *E1* and *E3*. Strains *E5* and *E6* both strains reached the stationary phase of growth at around day 9–10 (Additional file 1: Figure S4), but strain *E5* started decomposition of glucosinolates a few days earlier. Once the decomposition of SGN started, it ran with similar rate in the media of *E5* and *E6* (Table 3). The difference was more prominent for methylthioalkyl glucosinolates (Fig. 2b, Additional file 1: Figure S2e-f) and aromatic glucosinolates (Fig. 2c, Additional file 1: Figure S2 g-h). By the end of the study, *E6* decreased the concentration of thiomethylalkyl glucosinolates only, but the decrease was not significant (*p* > 0.05), in contrary to *E5* (*p* < 0.001). On the other hand, *E6* decomposed gluconasturtiin at 1.72-fold speed compared to *E5* [Table 3]. Strain *E4* showed a behavior similar to *E5-E6*, especially for methylthioalkyl and aromatic GSLs (Fig. 2b-c, Additional file 1: Figure S2e-h). It decomposed GSLs only after reaching the stationary phase of development. Strain *E2* was much more effective in the decomposition of aliphatic GSLs than the slow growing strains *E4–6* (Additional file 1: Figure S2b-d). However, these fungi degraded aromatic and methylthioalkyl GSLs with similar efficacy (Fig. 2b-c, Additional file 1: Figure S2e-h).

**Table 3:** Summary of the tested parameters of endophytic fungi isolated from horseradish and soil fungi

	Sole C source^a^	Glucosinolate decomposition in horseradish extract Residual amount after 16d (%)^b^				Decomposition rate in horseradish extract (mM d^-1^)				IC_50_ liquid culture (mM)	
ID	SGN	SGN	GIB	GLN	GBR	SGN	GIB	GLN	GBR	AITC	PEITC
*E1*	+	0.2 ± 0%	3.7 ± 0.9%	0 ± 0%	17 ± 3.1%	1.125	0.006	0.057	0.005	0.2834	0.0903
*E2*	+	0.3 ± 0%	25.6 ± 1.9%	0.4 ± 0.1%	28.1 ± 1.6%	1.476	0.002	0.033	0.004	0.2590	0.1004
*E3*	+	3.6 ± 0.8%	7.4 ± 0.9%	0.5 ± 0.2%	6.5 ± 1%	0.606	0.003	0.030	0.008	0.1925	0.0907
*E4*	+	0.3 ± 0%	36.3 ± 12.2%	0.4 ± 0.3%	33.9 ± 11.7%	0.734	0.003	0.036	0.006	0.1096	0.0288
*E5*		16 ± 18.9%	85 ± 10.3%	50.5 ± 14.8%	71.6 ± 8.1%	0.624	0.001	0.018	0.003	0.0920	0.0404
*E6*		0.2 ± 0.1%	42.5 ± 6.1%	3.3 ± 1.4%	26.6 ± 4.6%	0.783	0.001	0.032	0.006	0.0764	0.0328
*E7*		110.9 ± 4.9%	103.5 ± 4.4%	108.1 ± 2.3%	92.5 ± 10.1%	-	-	-	-	0.2565	0.0257
*S1*	+	0.1 ± 0%	5.1 ± 0.2%	0 ± 0%	65.3 ± 3.7%	3.037	0.007	0.214	0.008	0.1084	0.1511
*S2*	+	0.1 ± 0%	0 ± 0%	0 ± 0%	0 ± 0%	2.111	0.015	0.148	0.030	0.0743	0.0266
*S3*	+	85.2 ± 4.9%	75 ± 1.9%	74.8 ± 0.6%	83.9 ± 1%	-	-	-	-	0.2639	0.0700
*S4*		58.7 ± 5.7%	72 ± 5%	33.4 ± 5.7%	47.3 ± 4.7%	-	-	-	-	0.0338	0.0246
*S5*		87.5 ± 18.2%	101.4 ± 4.6%	106.7 ± 3.8%	98.7 ± 2%	-	-	-	-	0.1251	0.0176
*S6*		67.3 ± 12.1%	61.8 ± 9.4%	67.4 ± 14.4%	63.2 ± 10.4%	-	-	-	-	0.0277	0.0187
*S7*		105.2 ± 4.9%	90.3 ± 3.4%	95.7 ± 3.6%	89.8 ± 2.9%	-	-	-	-	0.0930	0.0738
*S8*		100.8 ± 2.5%	88 ± 4.8%	87.9 ± 4%	92.2 ± 3.5%	-	-	-	-	0.0867	0.0149
*S9*		0.3 ± 0.2%	0.1 ± 0.2%	0.2 ± 0.1%	0.4 ± 0.1%	2.982	0.012	0.206	0.018	0.0221	0.0553
*S10*		0.1 ± 0.1%	0.1 ± 0.1%	0.1 ± 0%	0.2 ± 0%	2.669	0.021	0.178	0.032	0.1954	0.1393

^a^Species marked by '+' accepted sinigrin as the sole carbon source

^b^Data for other glucosinolates is available in Additional file 3: Table 2

Decomposing ability was tested in pure horseradish extract as the growth medium. IC_50_ values of the isothiocyanates of horseradish against growth of fungi are given in end-concentration in the medium (mM). Fungi: *E1*, *Fusarium oxysporum; E2*, *Macrophomina phaseolina; E3*, *Fusarium oxysporum; E4*, *Setophoma terrestris; E5*, *Paraphoma radicina; E6*, *Paraphoma radicina; E7*, *Oidiodendron cerealis; S1-S10: soil fungi from the same site as one of the horseradish samples*

Abbreviations: *AITC* allyl isothiocyanate, *GBR* glucobrassicin, *GIB* glucoiberin, *GLN* gluconasturtiin, *n.d.* not determined, *PEITC* 2-phenylethyl-isothiocyanate, *SGN* sinigrin

The differences between decomposition rates of different classes of glucosinolates is also apparent from these data. The indolic GSLs were shown to be less prone to fungal decomposition (Fig. 2d, Additional file 1: Figure S2i-j), even though they were present at much lower concentrations than the main compounds. The other classes (aliphatic, methythiolalkyl and aromatic) were decomposed with more or less similar efficacy.

**Fig. 3:**
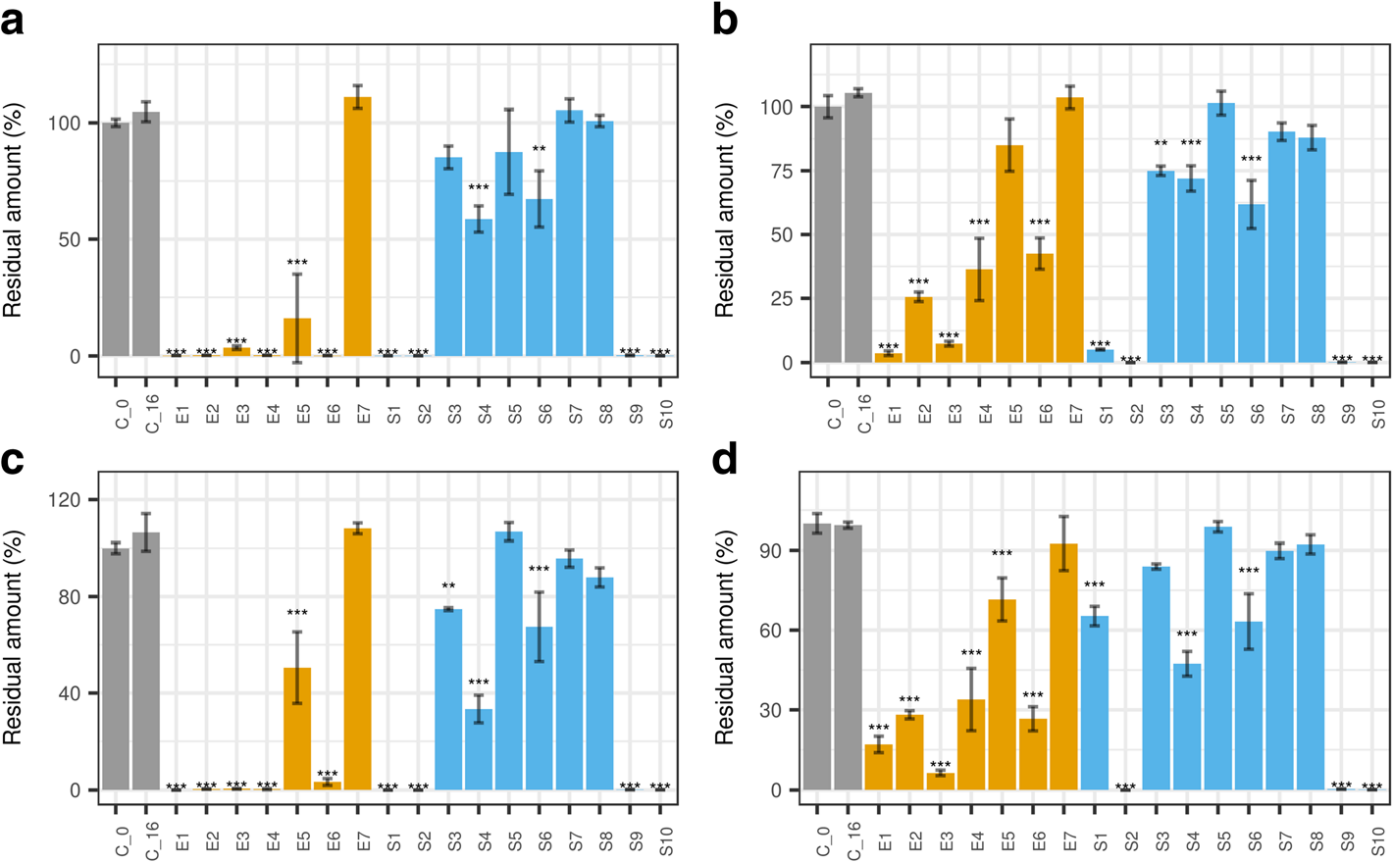
Decomposition of glucosinolates in horseradish extract incubated with endophytic and soil fungi for 16 days. Data are given for a single vial (equivalent to 0.295 g FW horseradish root). Subplots: a., sinigrin; b., glucoiberin; c., gluconasturtiin; d., glucobrassicin. Fungi: *E1*, *Fusarium oxysporum*; *E2*, *Macrophomina phaseolina*; *E3*, *Fusarium oxysporum*; *E4*, *Setophoma terrestris*; *E*5, *Paraphoma radicina*; *E6*, *Paraphoma radicina*; *E*7, *Oidiodendron cerealis*; *C_0*, control (zero time); *C_16*, control (end-time); *S1-S10: soil fungi from the same site as one of the horseradish samples.* Statistical test: Dunnett’s test, end-time samples were compared to end-time control (*n* = 3, ***, *p* < 3*10^− 6^; **, *p* < 3*10^− 5^ *, *p* < 1.9*10^− 4^). For the data in Table form, see Additional file 2 Table S2

The glucosinolate decomposing ability was less prevalent in the tested set of soil fungi. After the same 16-day incubation and sample preparation, the HRE samples were subjected to LC-MS for glucosinolate screening. The results showed that while some soil fungi were active, their overall efficacy was lower than that of the endophytes (Fig. 3, Additional file 1: Figure S5, Table 3, Additional file 2: Table S2). Four of ten strains decomposed all sinigrin by the end of the incubation period (*S1–2*, *S9–10*); two were only weak decomposers (*S4*, *S6*) and four could not metabolize sinigrin (*S3*, *S5*, *S7–8*).

Decomposition of other aliphatic GSLs was somewhat less efficient (Additional file 1: Fig. 5c-d, Table 3, Additional file 2: Table S2). Despite being able to decompose sinigrin and aliphatic GSLs, *S1* could not efficiently decompose indolic GSLs (Fig. 3d, Additional file 1: Figure S5i-j, Additional file 2: Table S2). This is similar to the phenomena observed in the case of endophytes. The active soil fungi, on the other hand, showed somewhat higher decomposition rates than endophytes (Table 3).

Thioglucosidase/myrosinase enzymes of fungal origin have already been described before [17, 18, 26, 47]. Based on our results, it seems, that this enzymatic activity is quite widespread. The potential glucosinolate decomposing ability shown above is a fine example of how the microbial community can modify the plant metabolome in various ways. The results also highlight the importance of within-species variability when studying plant – microbe interactions, as also shown by [48]. The fate of the glucosinolates was unclear at this point, therefore we aimed to detect their volatile decomposition products by SPME-GC-MS.

### Detection of decomposition products

We succesfully detected allyl isothiocyanate during decomposition of sinigrin by endophytes growing on horseradish extract solidified with agar. An SPME-GC-MS approach was necessary. Also, the glutathione conjugate of the allyl isothiocyanate was detected from the media by LC-MS.

The LC-MS data from the HRE incubation were first screened for desulfated glucosinolates using a modified protocol of [49]. The sulfatase enzyme from *Helix pomatia* worked well on both pure glucosinolates and the HRE. The desulfo-GSLs gave characteristic peaks in positive ion mode LC-MS (not shown), but none of these products were present in the HRE incubated with the endophytic fungi.

**Fig. 4:**
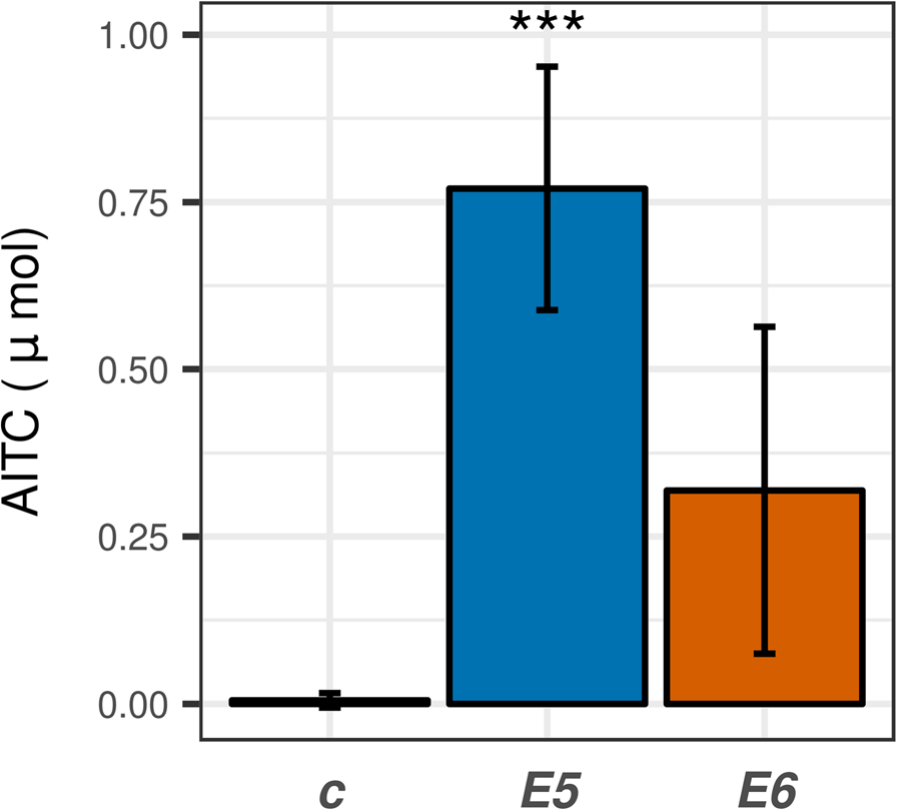
Allyl isothiocyanate content above the endophytic fungi growing on solidified horseradish extract. SPME was carried out on the 6th day for 24 h. The data were obtained by SPME-GC-MS, on day 6 (*n* = 3). Fungi: *E*5, *Paraphoma radicina*; *E6*, *Paraphoma radicina*; *C***,** control (not inoculated)*.* Statistical test: Dunnett’s test, 6th day samples compared to 6th day control (*n* = 3, ***, *p* < 10^− 3^; **, *p* < 10^− 2^ *, *p* < 0.05)

We also attempted to detect typical volatile decomposition products of sinigrin or gluconasturtiin, namely ITCs or nitriles. In the absence of specifier proteins or other special conditions, the decomposition products of glucosinolates are the isothiocyanates (Fig. 1) [13], which are highly toxic fungicides [15, 26]. Therefore the detection of isothiocyanates and the decomposition of glucosinolates infers myrosinase (thioglucosidase) activity in the fungi. Endophyte inoculation significantly influenced AITC concentration in the headspace as shown by GC-MS (Additional file 1: Figure S6a; *p* < 0.01). Surprisingly, only *E5* and *E6* caused a detectable release of AITC into the air of the Petri dish (Fig. 4, Additional file 1: Figure S6a). About 1.25% of the total amount of ITC was released into the air within 24 h on day 6 (*E6*). The other four SGN decomposing species (*E1*-*E4*) emitted trace amounts of AITC only. The nitrile generation would be a way to avoid toxicity of ITCs, and is shown in *Aspergillus* [17] where the product is nitrile. Despite this fact, allyl cyanide was not detected from any of the samples (Additional file 1: Figure S6b). No significant decomposition products of gluconasturtiin could be detected either (Additional file 1: Figure S6c), which could also be the result of their low volatility compared to AITC and allyl nitrile. The extracellular production of ITCs by *E5*–*6* can give these strains a competitive advantage over more sensitive species, as the released amount can result in concentrations inhibitory to fungi. However, ITCs are only detectable by GC-MS in their free form, the conjugates are not volatile. For example, despite that the liquid medium was directly extracted with organic solvent and analyzed by GC-MS, volatile products could not be detected during the rapid decomposition of sinigrin by *Citrobacter* [19]. It is possible that AITC was present in the medium only in conjugate form.

**Fig. 5:**
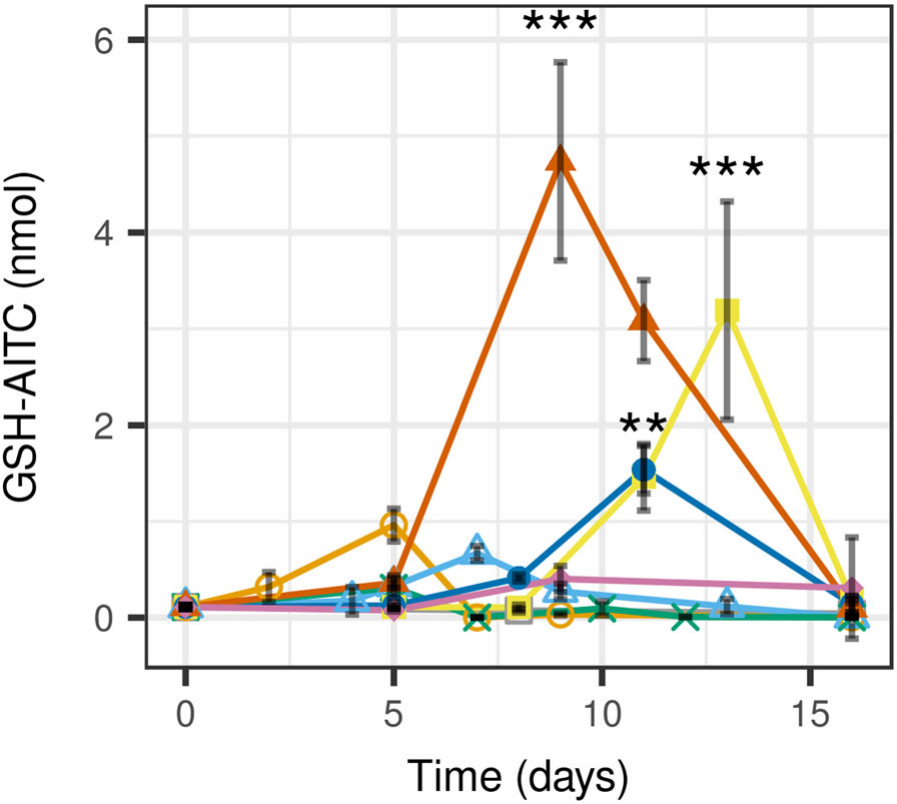
Change of concentration of a sinigrin metabolite, allyl isothiocyanate - glutathione adduct in horseradish extract inoculated by endophytic fungi from horseradish. Fungi: *E1*, *Fusarium oxysporum*; *E2*, *Macrophomina phaseolina*; *E3*, *Fusarium oxysporum*; *E4*, *Setophoma terrestris*; *E*5, *Paraphoma radicina*; *E6*, *Paraphoma radicina*; *E*7, *Oidiodendron cerealis*; *C*, control (not inoculated). Statistical test: Dunnett’s test, time points of highest concentrations (different for each strain) were compared to zero-time controls (*n* = 3, ***, *p* < 10^− 3^; **, *p* < 10^− 2^ *, *p* < 0.05)

As the volatile breakdown products were not found in case of many strains, a targeted search was run to find possible non-volatile GSL metabolites (CysGly – AITC, Cys – AITC, glutathione – AITC) from the LC-MS profiles was run. This approach revealed a peak with m/z 405.0914 at a retention time of 8.12 min. The compound was suspected to be GSH (glutathione) - AITC conjugate based on its exact mass. Its structure was confirmed by synthesizing the identical compound by mixing pure GSH and AITC in buffer, using the derivatization reaction employed in our recent study [33]. The two compounds showed identical MS^2^ spectra and retention times (Additional file 1: Figure S7). The GSH - AITC adduct concentration reached its maximum during the high-rate sinigrin decomposition for most strains (Figs. 2a, 5). The media of those fungi that did not release the AITC in the environment also contained high amounts of the GSH - AITC adduct.

Compared to zero-time control, the media of *E1* and *E2* contained 8.77 and 6.07-fold more GSH – AITC, respectively. This difference was much more expressed in the media of *E4*, *E5* and *E6* (29.14, 14.05 and 43.27-fold increase, respectively, *p* < 0.01, Dunnett’s test). *E3* did not cause any increase, which is likely to be the result of the flat decomposition curve for sinigrin (Figs. 2a, 5, Table 3). The presence of GSH - AITC adduct suggests that the ITCs are at least in part the decomposition products of GSLs in all fungi. AITC spontaneously conjugates with the thiol pool of the fungi (mainly GSH [50]) and is in part released into the medium. ITC toxicity increases when the GSH pool of the fungi becomes depleted [16], resulting in increased attack on the protein -SH groups. The rapid recovery of GSH from the conjugate could result in such a low detected amounts of GSH-AITC (Fig. 5). Recovery can be spontaneous or it can be catalyzed by glutathione-S transferase enzymes [51, 52]. The tolerance to AITC in the necrotrophic fungus *Alternaria brassicicola* was also characterized by elevation of glutathione-S transferases, and thioredoxins [27] The GSH-AITC can either be present in the medium as a result of active efflux or lysis of dead cells.

Even when GSH-AITC was detected, free GSH could also be detected from the medium in higher amounts. This suggest that the GSH pool was not depleted under the current conditions, and/or an active recovery mechanism is operating at high efficacy. The presence of free GSH also explains the observed viability of fungi during sinigrin decomposition. It is also possible that GSH is actively secreted into the medium, though only very limited extracellular functions of the molecule are described in fungi [50]. The high intracellular concentration and rapid recycling of GSH would also explain the findings of Albaser et al. [19]. They have shown that while the purified *Citrobacter* myrosinase decomposed pure glucosinolates into ITCs, AITC could not be detected from the medium of *Citrobacter* growing on sinigrin as the sole carbon source [19]. It is likely that the endophytic fungi of the current study behaved similarly to the mentioned *Citrobacter* strain during glucosinolate decomposition. Of course, production of a different, non-detected product or conjugate cannot be ruled out either.

Altogether, enzymes having thioglucosidase activity can be expected to be behind the observed GSL decomposition, as shown in a few instances [17, 18, 47]. The product was ITC, found either in free or conjugate form. Like the enzyme of *Citrobacter* examined by [19], these enzymes can also be adapted glucosidases (or homologues) that also accept thioglucosides as substrates. Figures 2 and 3 show that these microbial enzymes likely have wide specificity: they accept most glucosinolates as substrates, but the different GSLs are decomposed with different velocity. Different decomposition speeds against GSLs with different side-chains have also been described for plant myrosinases [33, 53] and the myrosinase of *Citrobacter* [19]. Despite that fungi seem to tolerate the released ITC quite well during growth on HRE, questions still remain. Decomposition does not necessarily mean the ability to selectively utilize the plant compounds. Therefore, growth on sinigrin as the sole carbon source was tested.

### The glucosinolate sinigrin as the sole carbon source for endophytic and soil fungi

Testing of the growth of fungi in media with sinigrin as the sole carbon source revealed that many strains have the ability to utilize the plant defensive compounds. In this case, a more marked difference between soil fungi and endophytes was found.

**Fig. 6:**
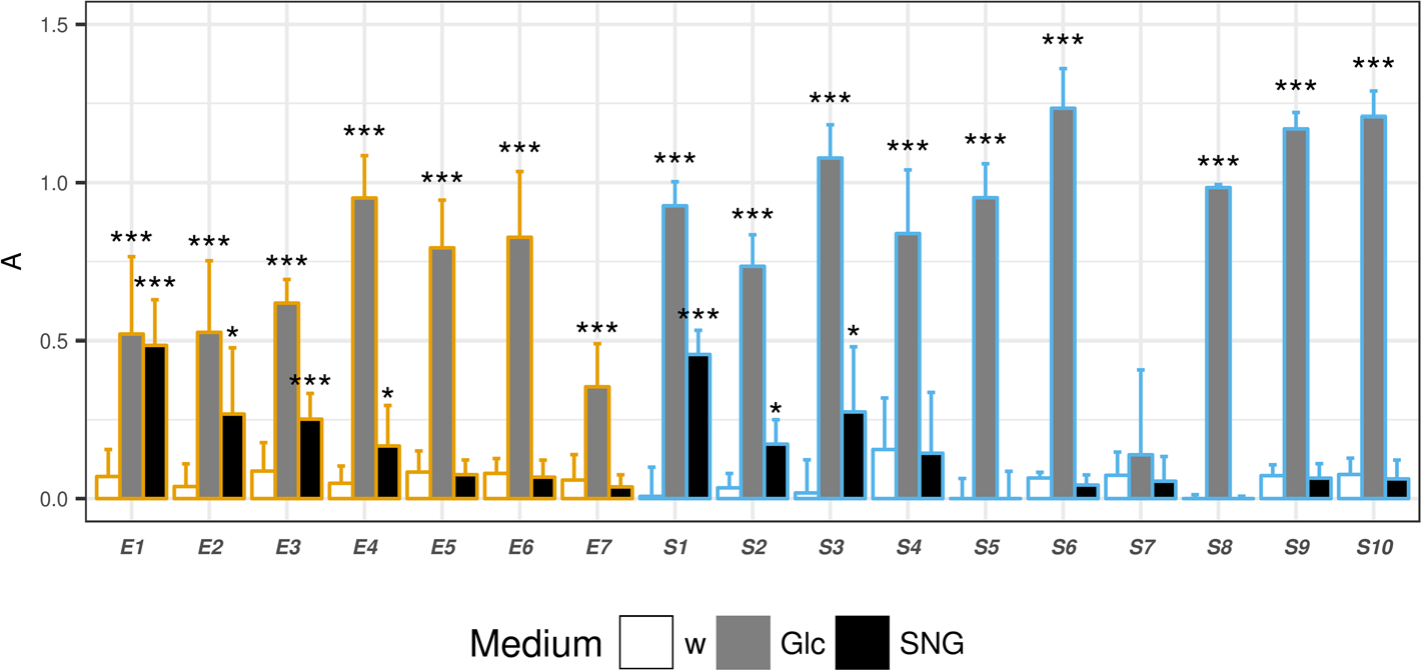
Growth of horseradish endophytes and soil fungi in Czapek Dox liquid media with different carbon sources. Data were obtained by measuring increased light absorbance (A) at 800 nm by a plate reader. Carbon sources: Glc, 2% glucose; SGN, sinigrin (equimolar to 2% glucose). Control is pure water (w). Fungi: *Fungi: E1, Fusarium oxysporum; E2, Macrophomina phaseolina; E3, Fusarium oxysporum; E4, Setophoma terrestris; E5, Paraphoma radicina; E6, Paraphoma radicina; E7, Oidiodendron cerealis*; *S1-S10: soil fungi from the same site as one of the horseradish samples.* Statistical test: Dunnett’s test, end-time samples were compared to end-time controls, for each fungus separately (*n* = 3, ***, *p* < 10^− 3^; **, *p* < 10^− 2^ *, *p* < 0.05)

Surprisingly, four of seven horseradish endophytes (*E1*–*4*) accepted sinigrin as the sole carbon source (*p* < 0.05). The most efficient sinigrin decomposer performed well on both sinigrin and glucose as carbon sources (*E1*, Figs. 2a, 6). Less efficient decomposers showed growth but apparently sinigrin provided less favorable conditions than glucose (*E2–4****,*** Fig. 6). This is another good example of within-species variability (*E1* vs *E3*).

When subjecting the soil fungi to the same experiment, only three of ten strains were capable of using sinigrin as the sole carbon source (*p* < 0.05, Fig. 6). Therefore this phenomenon seems to be more specific than GSL decomposition. Though the endophytes could not be clearly separated from the soil fungi by their GSL decomposition ability, the chance to find endophytes accepting sinigrin as the sole carbon source was 1.7-fold higher compared to that of the soil fungi. One strain could not even develop on the Czapek Dox medium (*S7*), perhaps due to the lack of a favorable organic N source. An active strain (*S1*) was found to belong to the same species as *E1*, *E3* based on morphology and its ITS sequence. One could think that *Fusarium* sp. tend to have some enzyme that decomposes GSLs, which may be an explanation behind the high prevalence of *Fusarium* strains in the root microbiome of Brassicaceae species [25]. However, *S3*, *S7* were also identified as *Fusarium* sp. based on ITS and eF1 sequences as well as their morphological characteristics. These strains showed much less glucosinolate decomposing potential (Fig. 3, Table 3, Additional file 1: Figure S5). The above highlight the significance of within-species variability in similar approaches. Therefore, more studies are required to fully explain the potential interaction between the GSLs and *Fusarium sp*. Other sinigrin-utilizing microbes have been described before, e.g. some strains of *Aspergillus sp*. [17, 54] and bacteria [19, 23, 55]. It is worth mentioning that this activity can be achieved by expressing a wide-specificity β-O-glucosidase as in the case of *Citrobacter* [19]. This might have been the case in the current study.

Evidently, we can state that the endophytes from horseradish roots are rich sources of enzymes that can break down the secondary metabolites of the host plant. Moreover, these strains are able to use the defensive metabolites as nutrients to develop, which ability is less abundant among the soil fungi. This is interesting in the light of the fact that these defensive metabolites give rise to antifungal compounds upon decomposition. The enzymes responsible for the breakdown of the glucosinolates can be specific thioglucosidases, or β-O-glucosidases with wider specificity as well. The above results suggest the adaptation of the endophytes to the defensive metabolites of the plant, which is highlighted by the higher chance to find active endophytes than soil fungi. After investigating the effects of the endophytes on the plant metabolites, the effects of the ITCs on the fungi were also examined, with special regard to the soil fungi – endophyte difference.

### Growth inhibition by isothiocyanates

Relatively low amounts of ITCs successfully inhibited the growth of all tested fungi in liquid media. The inhibition data with the fitted curves and IC_50_ calculation are shown in Additional file 1: Figures. S8 and S9. Again, a difference was found between the endophyte and the soil fungal group: the endophytes tolerated AITC better.

It is apparent from the IC_50_ values in Table 3 that most fungal strains were more tolerant to AITC than PEITC: the average difference between the IC_50_ values is 2.30-fold. The difference is likely to be a consequence of the different stability of the ITC adducts with proteins [51], as well as the different polarity and therefore different solubility in water, different kinetics of the ITCs themselves.

If we recall the AITC release data from the SPME-GC-MS experiment (Fig. 4), we get a theoretical 0.07 mM concentration for AITC in the liquid medium, assuming complete dissolution in the medium which is usually the case [56]. By comparing this with the IC_50_ data (Table 3), it is apparent that the AITC is released from horseradish extract by *E5* and *E6* is enough to theoretically inhibit growth of vulnerable strains (*S2*, *S6*, *S9*). The same amount would also slow the growth of *E5* and *E6* as well, perhaps that is an another reason why the GSL decomposing enzymes are not expressed until the end of growth.

By carefully comparing the distribution of the IC_50_ values of the two groups (endophytes vs soil fungi, Table 3), it can be observed that the endophytic group almost lacks fungi with high vulnerablity to AITC. Only two of seven endophytes had an IC_50_ below 0.1 mM, while more than half of the soil fungi fell in the same range. By directly comparing the median IC_50_ values, a two-fold advantage of the endophytes was recognized: the median IC_50_ values for soil fungi and endophytes were 0.090 and 0.192 mM AITC, respectively. This difference is not found in the case of PEITC: median IC_50_ values for soil fungi and endophytes were 0.040 and 0.041 mM, respectively. As the phenomenon is extremely overlaid by interspecies variability, further studies will be necessary to provide further evidence on this logically expectable adaptation to ITCs. The background mechanisms behind the adaptation likely include genes that enable enhanced recycling or excessive production of thiols [27], such as GSH [16, 50]. This can lead to resistance to fungicides that target the thiol pool of phytopathogenic fungi [57, 58].

The difference of the sensitivity of the fungi to the two tested ITCs is a fine example of the mechanism behind the plant’s ability to drive the development of its microbiome [59]. It also exemplifies the rationale behind producing various ITC precursors for chemical defense. Actually, the mixture of ITCs can limit activity of much more strains than AITC or PEITC alone would be capable of, without even taking synergistic effects into account [16].

## Conclusions

Our main conclusion is that endophytes are active participants of the glucosinolate – myrosinase – isothiocyanate system. The present work also provides preliminary data that suggest existence of a detectable adaptation of the endophytes to the host plant’s antifungal secondary metabolites. This adaptation distinguished the endophytic fungi from the soil fungi from the same soil to some extent. We used various experimental approaches to find different manifestations of this adaptation. These were the following: 1., The endophytes were more tolerant to the plant defensive compound allyl isothiocyanate than the soil fungi. 2., Compared to soil fungi, a higher proportion of endophytic strains were capable of glucosinolate decomposition. 3., Compared to soil fungi, a higher proportion of endophytes could utilize sinigrin as the sole carbon source. The results have shown that no clear boundary can be drawn between the root endophytes and soil fungi, but still, there is a detectable difference. The case is similar to that found when the ratio of new natural products from endophytes and soil fungi was compared [60]. The results also fit the endophytic continuum approach very well: endophytes can be present at many points along a functional gradient. The phenomena found at the same time show examples of mechanisms by which plants drive the development of their root microbiome by exudates. The root endophytes found are likely a subset of the available soil fungi that have the biochemical potential and opportunity to colonize the plant’s roots. Their competitive advantage came from the higher potential tolerance of the plant’s defensive compounds, among others. If the fungal myrosinase is expressed *in planta* while the plant is still healthy, this may be a possible mechanism by which endophytes decrease incidence of plant diseases in Brassicaceae species. The same would result in changes in the plant microbiome that would be controlled by endophytes by releasing plant defensive metabolites. It can also be a means by which the endophytes override plant chemical defense reactions. Using a myrosinase is a way to disarm the chemical bomb of glucosinolates that the fungus will encounter upon infection of the plant root. As many other interesting possibilities exist, we think that the myrosinase activity and the chemical adaptation of endophytes warrants further studies.

## Additional files

Additional file 1: **Figures S1-S9.** Supplementary Figures (including Compound structures; Decomposition of minor glucosinolates in horseradish extract by endophytic and soil fungi; Sinigrin decomposition by endophytes in sinigrin-supplemented Saboraud Glucose Broth; Growth curves; SPME-GC-MS chromatograms; LC-MS extracted ion chromatograms; Growth inhibition curves and IC_50_ calculation) and additional methodical details on SPME-GC-MS. (PDF 5696 kb)

Additional file 2: **Table S1.** Changes in concentration of glucosinolates in horseradish extracts incubated with horseradish endophytes. Raw abundance data. (DOC 58 kb)

Additional file 3: **Table S2.** Residual amount of sinigrin and minor glucosinolates in horseradish extract after 16 days of incubation with endophytic fungi from horseradish and soil fungi. (DOC 33 kb)

## Abbreviations

AITC: allyl isothiocyanate
CzD: Czapek Dox medium
GC-MS: Gas Chromatography - Mass Spectrometry
GSH: Glutathione, reduced form
GSL: Glucosinolate
HRE: horseradish extract
ITC: Isothiocyanate
LC-MS: High Performance Liquid Chromatography - Mass Spectrometry
MYR: myrosinase
PDA: Potato dextrose agar
PEITC: 2-phenylethyl isothiocyanate
PES: Polyethersulfone membrane
SGA: Saboraud Glucose Agar
SPME: Solid phase micro extraction
