# Supplementary material for: Endophytic fungi from the roots of horseradish (Armoracia rusticana) and their interactions with the defensive metabolites of the glucosinolate - myrosinase - isothiocyanate system
Source: BMC Plant Biol. 2018 May 9;18:85. doi: 10.1186/s12870-018-1295-4 (PMC5944135; doi:10.1186/s12870-018-1295-4)
Supplement: Supplementary file 1 — Figures S1-S9. Supplementary Figures (including Compound structures; Decomposition of minor glucosinolates in horseradish extract by endophytic and soil fungi; Sinigrin decomposition by endophytes in sinigrin-supplemented Saboraud Glucose Broth; Growth curves; SPME-GC-MS chromatograms; LC-MS extracted ion chromatograms; Growth inhibition curves and IC50 calculation) and additional methodical details on SPME-GC-MS. (PDF 5696 kb) [file 12870_2018_1295_MOESM1_ESM.pdf]

Supplementary material

SPME-GC-MS using activated charcoal as adsorbent – some technical details

We optimized an SPME method to detect the most frequent metabolites. Automated sampling in headspace vials gives the opportunity to sample a few cm^2 surface only, which resulted in limited detectability. Therefore, endophytes were inoculated on solidified horseradish extract in Petri dishes. In these dishes, powdered charcoal (Ph. Eur.) was placed as adsorbent in the air. Using aqueous solutions of allyl isothiocyanate, the amount of charcoal per plate and the elution solvent was optimized. The method was subsequently tested for other analytes of interest (allyl cyanide, phenylpropionitrile, phenethyl isothiocyanate). The optimal amount of charcoal was 5 mg, higher amounts (10, 25, 50 mg) gave significantly smaller peak areas for AITC. Ethyl acetate was much better for elution than hexane or tert-butyl-methyl-ether.

To be able to detect allyl nitrile, ethyl acetate had to be replaced with a solvent that has similar polarity but significantly lower boiling point. Analysis of allyl cyanide required the initial GC oven temperature to be set as low as 35 ºC. Methyl acetate (or ethyl formate) was found to be most suitable, therefore it was used during the rest of the study.

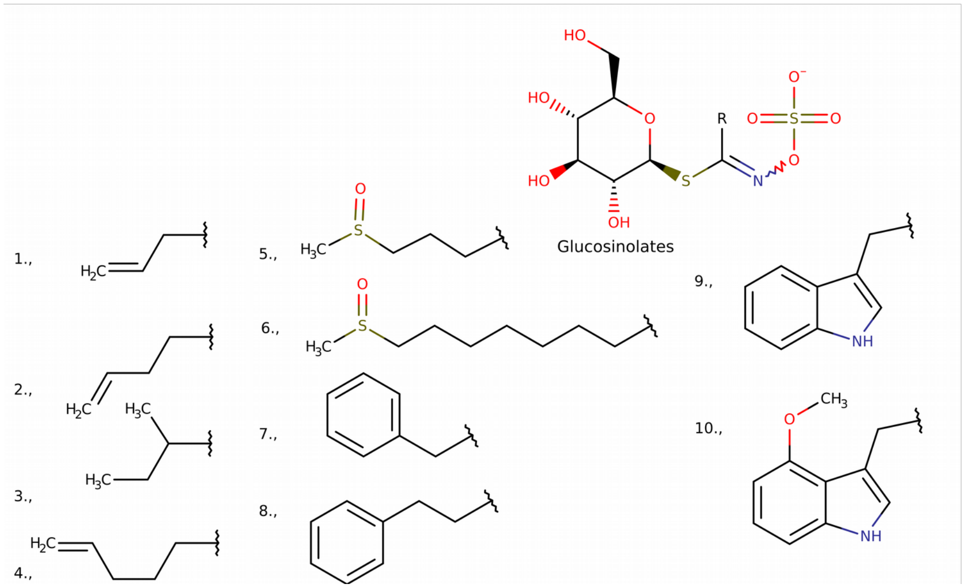

Figure S1.

Generic structure of glucosinolates and side chains of the glucosinolates tested for decomposition by horseradish endophytes in the current study. Chromatographic parameters of these analytes can be found in Table 2. Glucosinolates: 1: Sinigrin, 2: Gluconapin, 3: Glucocochlearin, 4: Glucobrassicanapin, 5: Glucoiberin, 6: Glucoibarin, 7: Glucotropaeolin, 8: Gluconasturtiin, 9: Glucobrassicin, 10: 4-Methoxyglucobrassicin. Numbering corresponds to that in Table 2., and subplots a-j. (respectively) in Supplementary Fig. 2.

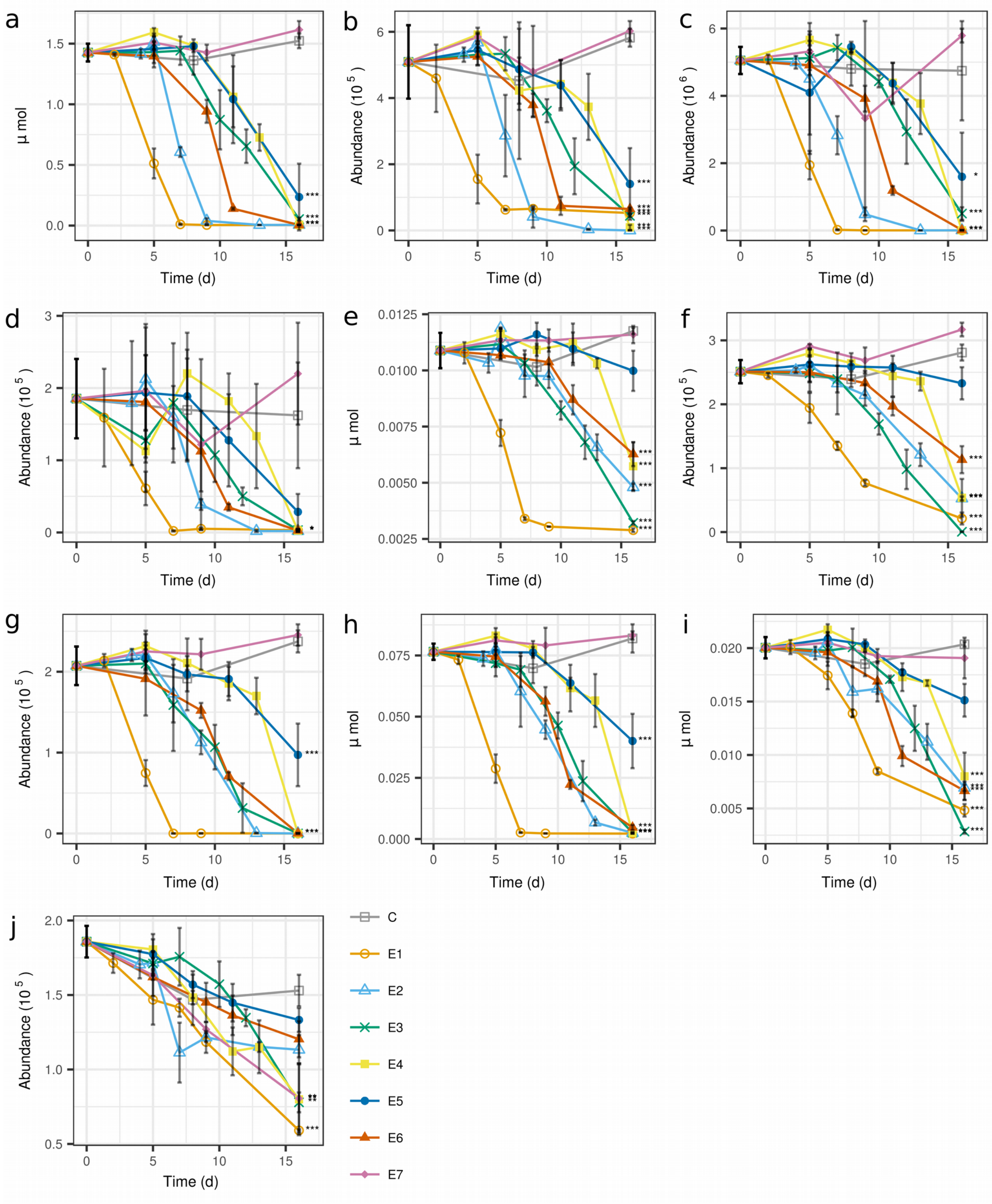

Figure S2.

Decomposition of glucosinolates in horseradish extract inoculated by endophytic fungi from horseradish. The following glucosinolate subclasses are presented: a-d., aliphatic; e-f, methylthioalkyl; g-h, aromatic; i-j, indolic. Subplots: a., sinigrin; b., gluconapin; c., glucocochlearin; d., glucobrassicanapin; e., glucoiberin; f., glucoibarin; g., glucotropaeolin; h., gluconasturtiin; i., glucobrassicin; j., 4-methoxyglucobrassicin. Fungi: *E1*, *Fusarium oxysporum*; *E2*, *Macrophomina phaseolina*; *E3*, *Fusarium oxysporum*; *E4*, *Setophoma terrestris*; *E5*, *Paraphoma radicina*; *E6*, *Paraphoma radicina*; *E7*, *Oidiodendron cerealis*; *C*, control (not inoculated). Statistical test: Dunnett's test, endtime samples compared to end-time control (n = 3, ***, $p < 10^{-5}$; **, $p < 10^{-4}$ *, $p < 5*10^{-4}$).

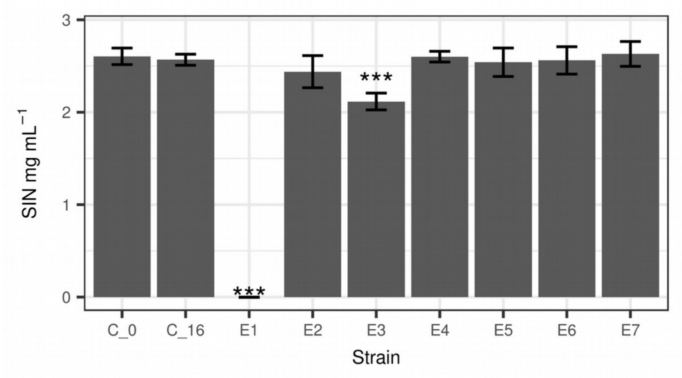

Figure S3.

Residual sinigrin in Saboraud Glucose Broth supplemented with 2.5 mg mL^{-1} sinigrin (comparable to that found in the horseradish extract) after incubation with endophytic fungi from horseradish for 16 days. Legend: C_0, zero time control; C_16, endtime control; Endophytic fungi (16 days): *E1*, *Fusarium oxysporum; E2*, *Macrophomina phaseolina; E3*, *Fusarium oxysporum; E4*, *Setophoma terrestris; E5*, *Paraphoma radicina; E6*, *Paraphoma radicina; E7*, *Oidiodendron cerealis.* Applied statistical test was ANOVA followed by Dunnett's test, '***', $p < 0.001$.

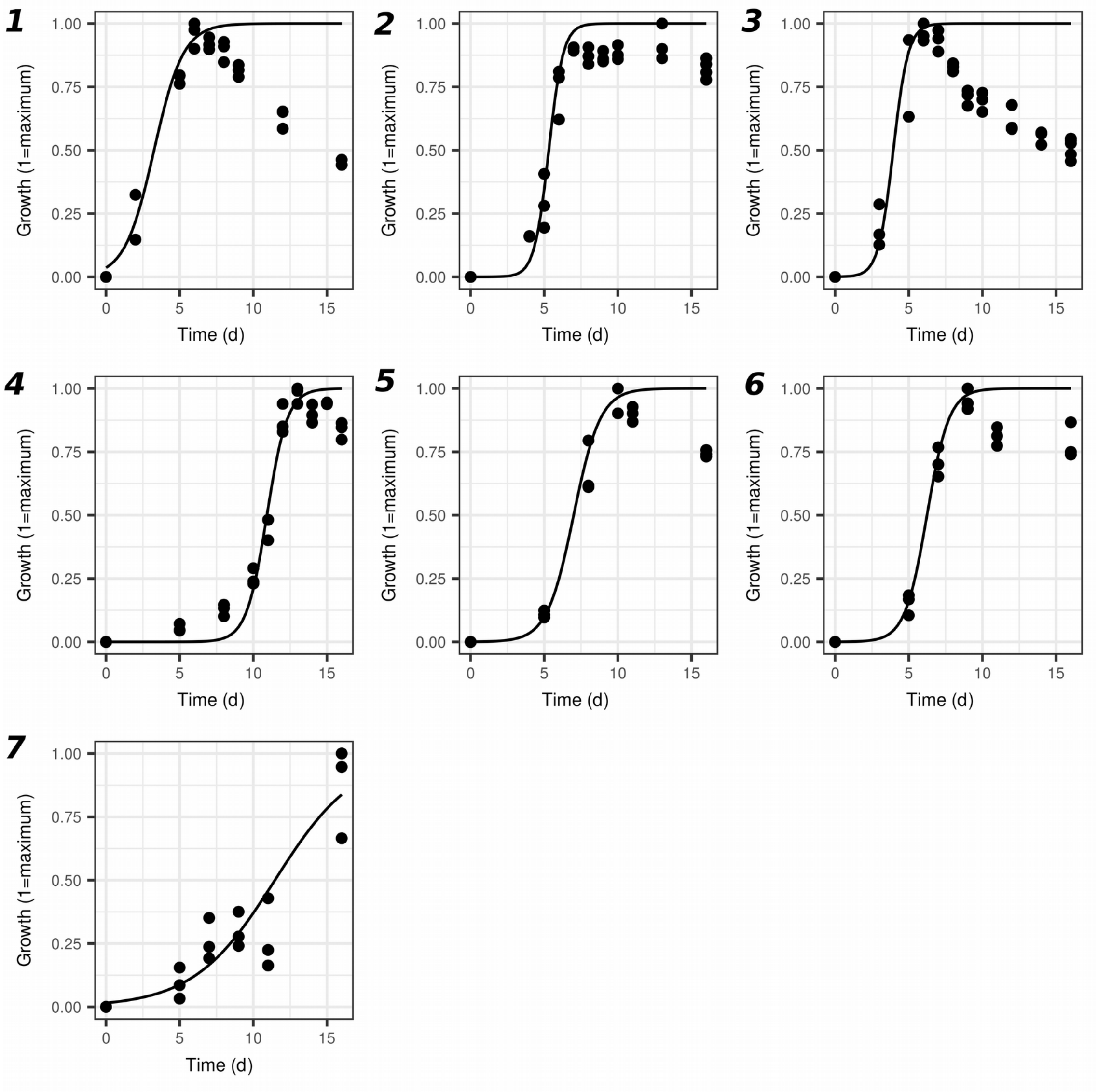

Figure S4.
Growth of fungal endophytes from horseradish in the glucosinolate-rich horseradish extract. The plots show the normalized dry weight versus incubation time, and the growth curve fitted to the data (an average of at least 20 fit attempts). Subplots show data for individual fungi, as follows: *E1*, *Fusarium oxysporum*; *E2*, *Macrophomina phaseolina*; *E3*, *Fusarium oxysporum*; *E4*, *Setophoma terrestris*; *E*5, *Paraphoma radicina*; *E6*, *Paraphoma radicina*; *E*7, *Oidiodendron cerealis*.

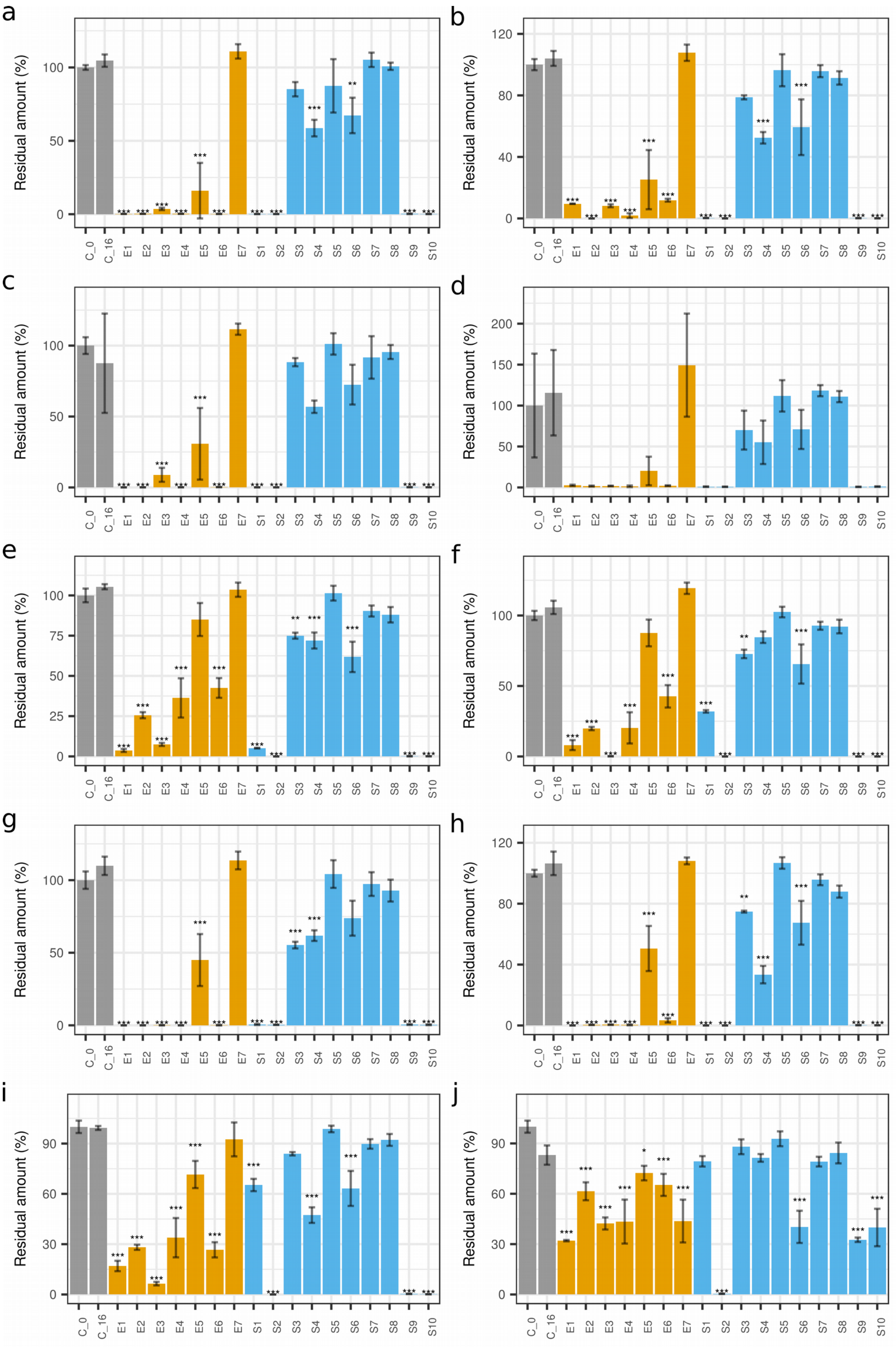

Figure S5.

Decomposition of sinigrin and minor glucosinolates in horseradish extract after 16 days of incubation with endophytic fungi from horseradish and soil fungi. The following glucosinolate subclasses are presented: a-d., aliphatic; e-f, methylthioalkyl; g-h, aromatic; i-j, indolic. <u>Subplots</u>: a., sinigrin; b., gluconapin; c., glucocochlearin; d., glucobrassicanapin; e., glucoiberin; f., glucoibarin; g.,

glucotropaeolin; h., gluconasturtiin; i., glucobrassicin; j., 4-methoxyglucobrassicin. Fungi: *E1*, *Fusarium oxysporum*; *E2*, *Macrophomina phaseolina*; *E3*, *Fusarium oxysporum*; *E4*, *Setophoma terrestris*; *E5*, *Paraphoma radicina*; *E6*, *Paraphoma radicina*; *E7*, *Oidiodendron cerealis; C_0*, control (zero time); *C_16*, control (end-time); *S1-S10: soil fungi from the same site as one of the horseradish samples.* Statistical test: Dunnett's test, end-time samples were compared to end-time control (n = 3, ***, $p < 3*10^{-6}$; **, $p < 3*10^{-5}$ *, $p < 1.9*10^{-4}$).

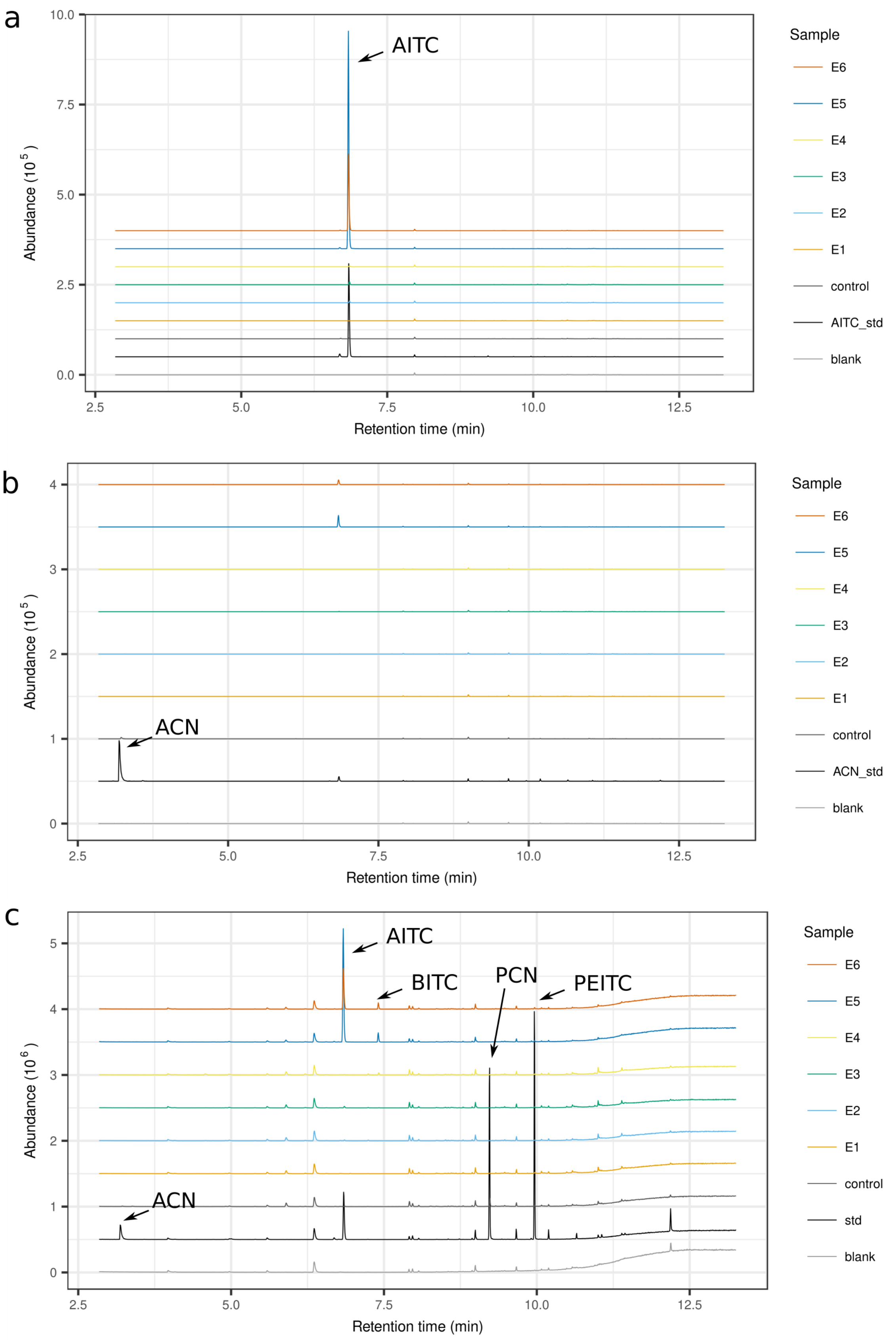

Figure S6.

GC-MS traces of volatiles released from horseradish extract by endophytic fungi during growth on solidified horseradish extract. Typical replicates are presented. Split ratio was 10:1, other parameters are detailed in the main text. Volatile constituents were adsorbed on activated charcoal from the air of

the agar plate for 24 hours and subsequently eluted with methyl acetate for GC-MS analysis.

Abbreviations: ACN, allyl nitrile; AITC, allyl isothiocyanate; BITC, butyl isothiocyanate isomer (m/z 115, likely from glucocochlearin); PCN, phenypropionitrile; PEITC, 2-phenylethyl isothiocyanate.

Subplots: a., Allyl isothiocyanate (SIM m/z 99); b., Allyl cyanide (SIM m/z 67); c., total ion chromatogram (TIC).

Legend: blank, a charcoal eluate (nothing adsorbed); AITC_Std, 100 μg mL^{-1} allyl isothiocyanate (authentic standard); ACN_std, 100 μg mL^{-1} allyl nitrile (authentic standard); control, horseradish extract with no fungal inoculation; *E1-E6*: endophytic fungi from horseradish (on days of maximal abundances); std, Standard mixture containing allyl cyanide, allyl isothiocyanate, phenylpropionitrile and 2-phenylethyl isohtiocyanate (100 μg mL^{-1} each).

Color code of fungi matches that of Fig.2. Fungal samples: *E1*, *Fusarium oxysporum* (day 5); *E2*, *Macrophomina phaseolina* (day 7); *E3*, *Fusarium oxysporum* (day 6); *E4*, *Setophoma terrestris* (day 6); *E*5, *Paraphoma radicina* (day 6); *E6*, *Paraphoma radicina* (day 6).

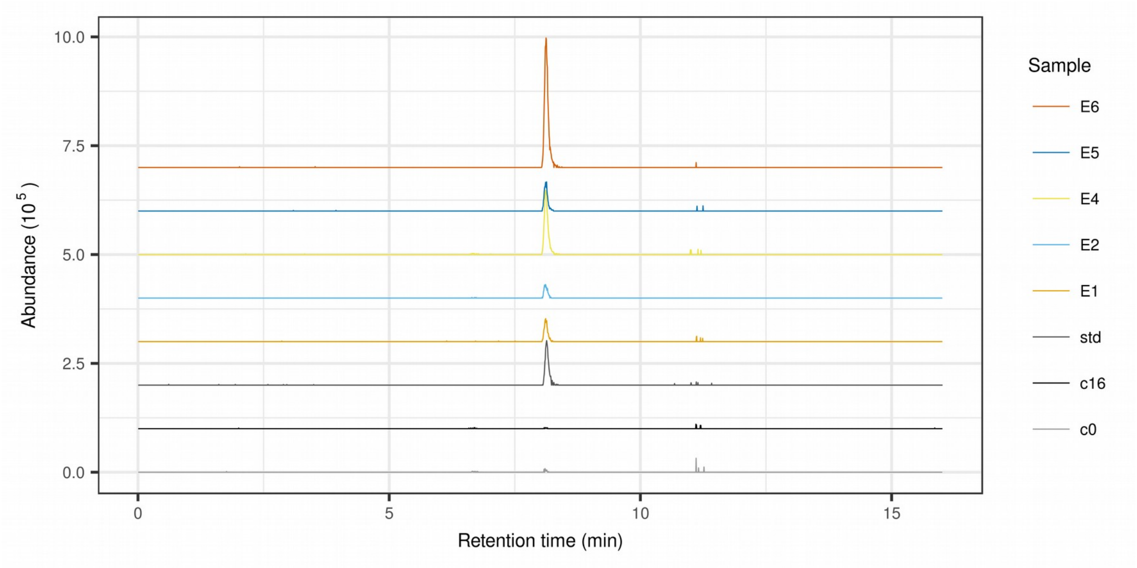

Figure S7.
LC-MS traces of GSH-AITC adduct, a metabolite of sinigrin detected in the horseradish extract during growth of endophytic fungi of horseradish. The trace of m/z 405.090 ($[M-H]^-$) is shown. Accuracy was ± 5 ppm. Legend: c0, zero time control; c16, endtime control; std, GSH-AITC adduct formed by reacting GSH and AITC in buffer (equivalent to 0.001 µg mL^{-1} AITC); *E1-E6*: endophytic fungi from horseradish at the maximal detected amount of GSH-AITC adducts (on days of maximal abundances). Color code of fungi matches that of Supplementary Fig. 2. Fungi: *E1*, *Fusarium oxysporum* (day 5); *E2*, *Macrophomina phaseolina* (day 7); *E4*, *Setophoma terrestris* (day 13); *E*5, *Paraphoma radicina* (day 11); *E6*, *Paraphoma radicina* (day 9).

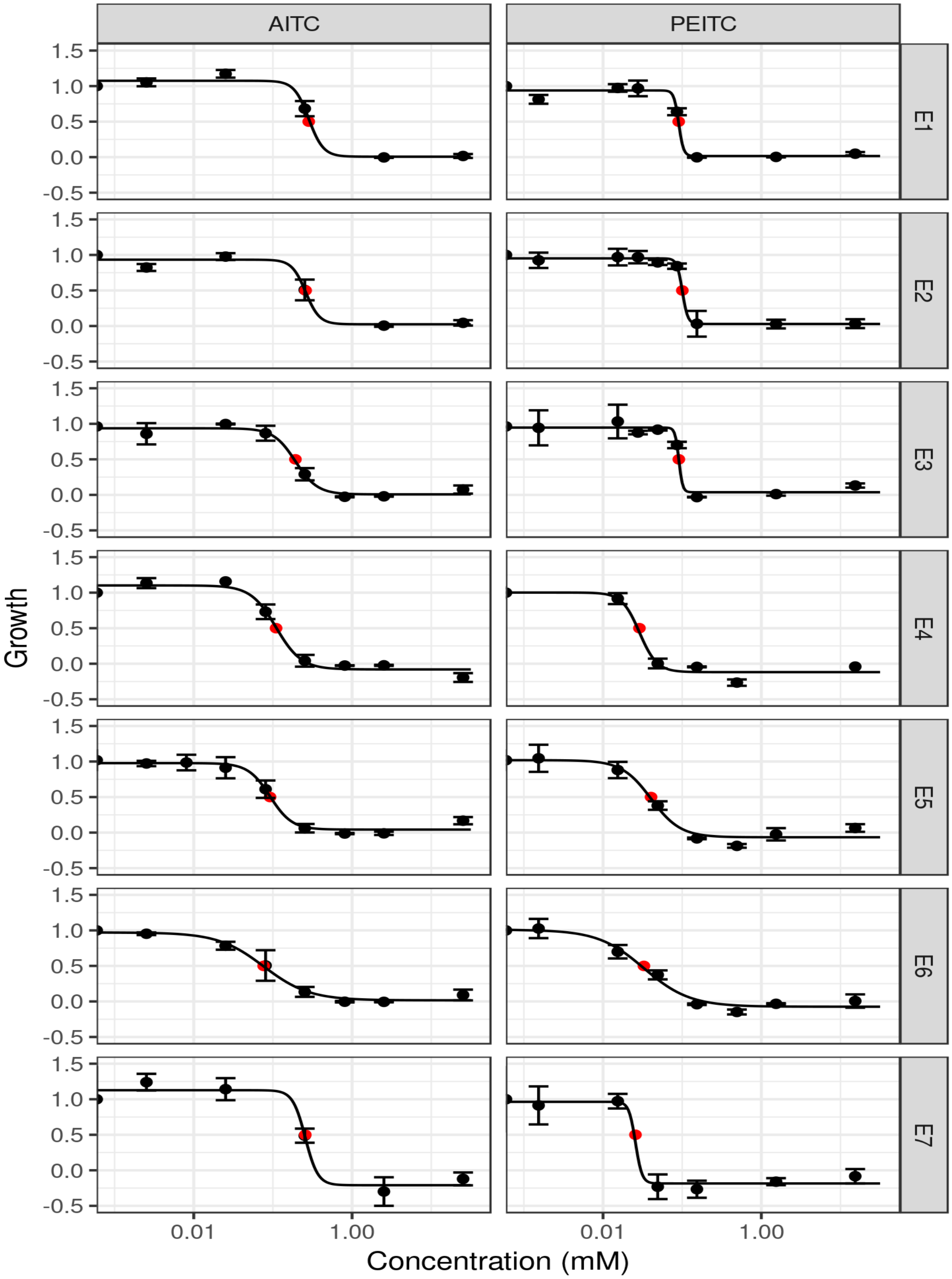

Figure S8.

Growth inhibition curves of endophytic by horseradish isothiocyanates allyl isothiocyanate (AITC) and 2-phenylethyl isothiocyanate (PEITC). These points were used to fit curves to obtain IC_{50} concentrations of isothiocyanates (shown in red). *E1-E7* (indicated on the right): endophytic fungi from horseradish.

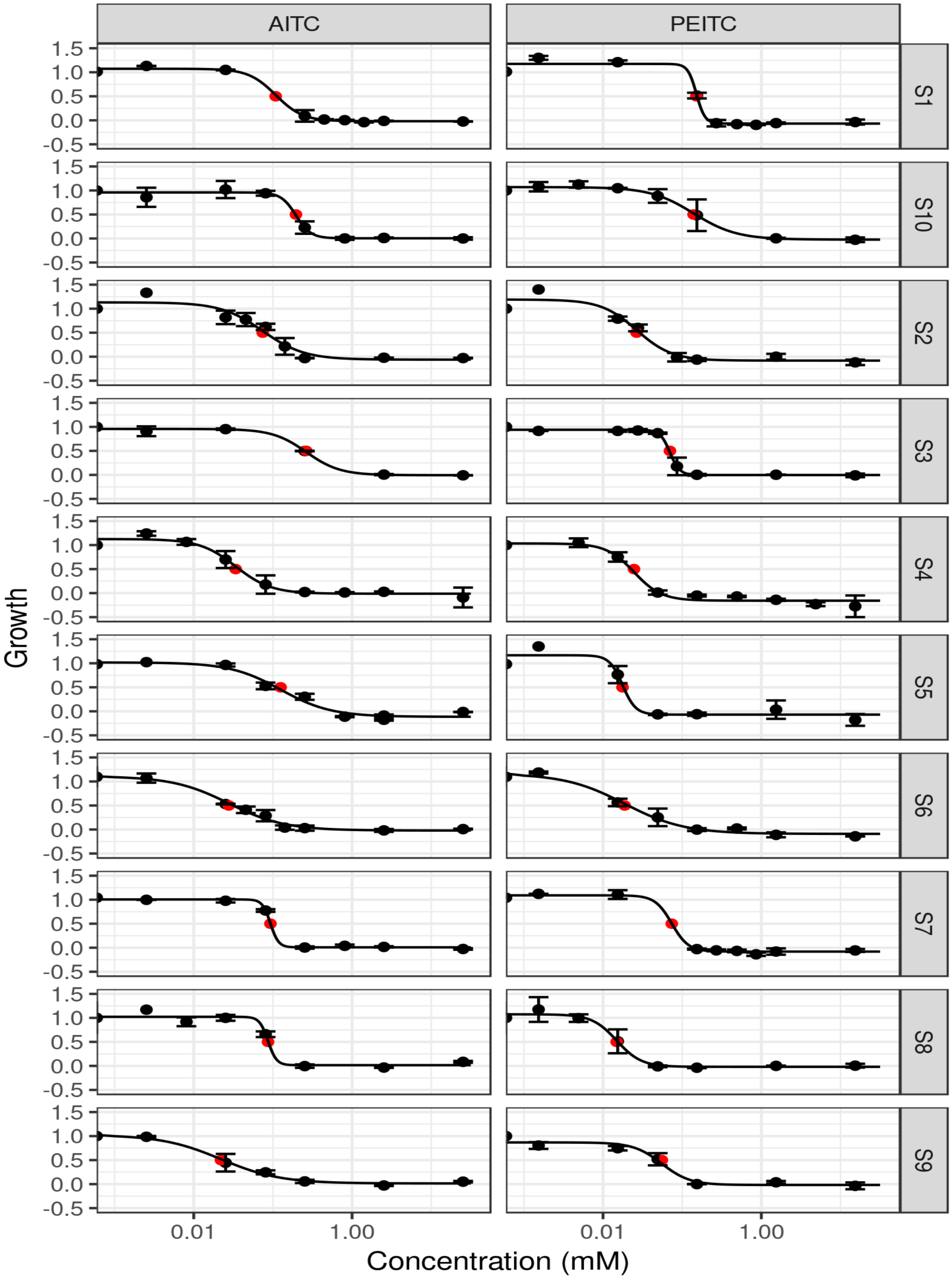

Figure S9.

Growth inhibition curves of soil by horseradish isothiocyanates allyl isothiocyanate (AITC) and 2-phenylethyl isothiocyanate (PEITC). These points were used to fit curves to obtain IC_{50} concentrations of isothiocyanates (shown in red). *S1 – S10* (indicated on the right): soil fungi from the same soil as one of the horseradish samples.
