# Supplementary material for: Endophytic fungi from the roots of horseradish (Armoracia rusticana) and their interactions with the defensive metabolites of the glucosinolate - myrosinase - isothiocyanate system
Source: BMC Plant Biol. 2018 May 9;18:85. doi: 10.1186/s12870-018-1295-4 (PMC5944135; doi:10.1186/s12870-018-1295-4)
Supplement: Supplementary file 2 — Table S1. Changes in concentration of glucosinolates in horseradish extracts incubated with horseradish endophytes. Raw abundance data. (DOC 58 kb) [file 12870_2018_1295_MOESM2_ESM.doc]

**Table S1.**

Changes in concentration of glucosinolates in horseradish extracts incubated with horseradish endophytes. Raw abundance data (area under curve) are shown. Data were obtained using XCMS Online, using parameters suggested for Orbitrap, with minor modifications. Treatments: all, all treatments (including controls); C, control (no fungal inoculation); *E1-7*: endophytic fungi: *E1*, *Fusarium oxysporum*; *E2*, *Macrophomina phaseolina*; *E3*, *Fusarium oxysporum*; *E4*, *Setophoma terrestris*; *E*5, *Paraphoma radicina*; *E6*, *Paraphoma radicina*; *E*7, *Oidiodendron cerealis*;

| Time | Treat-  ment | Sinigrin | Gluconapin | Gluco-cochlearin | Gluco- brassicanapin | Glucoiberin | Glucoibarin | Gluco-tropaeolin | Gluco-nasturtiin | Gluco-brassicin | 4-Methoxy-gluco-brassicin |
| --- | --- | --- | --- | --- | --- | --- | --- | --- | --- | --- | --- |
| 0 | all | 3.146 ± 0.162 E8 | 5.090 ± 1.110 E5 | 5.051 ± 0.404 E6 | 1.853 ± 0.549 E5 | 1.648 ± 0.156 E6 | 2.511 ± 0.182 E5 | 2.073 ± 0.238 E5 | 3.404 ± 0.152 E7 | 5.399 ± 0.292 E6 | 1.858 ± 0.106 E5 |
| 2 | *E1* | 3.108 ± 0.022 E8 | 4.595 ± 1.018 E5 | 5.088 ± 0.138 E6 | 1.590 ± 0.677 E5 | 1.627 ± 0.094 E6 | 2.454 ± 0.038 E5 | 2.137 ± 0.083 E5 | 3.253 ± 0.081 E7 | 5.417 ± 0.194 E6 | 1.715 ± 0.066 E5 |
| 4 | *E2* | 3.124 ± 0.087 E8 | 5.310 ± 0.216 E5 | 4.996 ± 0.188 E6 | 1.791 ± 0.859 E5 | 1.539 ± 0.086 E6 | 2.531 ± 0.040 E5 | 2.206 ± 0.076 E5 | 3.274 ± 0.137 E7 | 5.255 ± 0.174 E6 | 1.705 ± 0.093 E5 |
| 5 | *E1* | 1.127 ± 0.272 E8 | 1.552 ± 0.737 E5 | 1.941 ± 0.423 E6 | 6.095 ± 2.321 E4 | 9.210 ± 1.150 E5 | 1.943 ± 0.235 E5 | 7.474 ± 1.598 E4 | 1.217 ± 0.266 E7 | 4.639 ± 0.379 E6 | 1.468 ± 0.167 E5 |
| 5 | *E2* | 3.341 ± 0.149 E8 | 5.674 ± 0.263 E5 | 4.506 ± 1.658 E6 | 2.124 ± 0.715 E5 | 1.846 ± 0.006 E6 | 2.611 ± 0.256 E5 | 2.203 ± 0.073 E5 | 3.446 ± 0.107 E7 | 5.577 ± 0.238 E6 | 1.717 ± 0.071 E5 |
| 5 | *E3* | 3.153 ± 0.199 E8 | 5.313 ± 0.314 E5 | 5.123 ± 0.512 E6 | 1.277 ± 0.713 E5 | 1.704 ± 0.136 E6 | 2.483 ± 0.287 E5 | 2.101 ± 0.120 E5 | 3.189 ± 0.246 E7 | 5.319 ± 0.417 E6 | 1.711 ± 0.084 E5 |
| 5 | *E4* | 3.514 ± 0.077 E8 | 5.916 ± 0.208 E5 | 5.662 ± 0.163 E6 | 1.128 ± 0.160 E5 | 1.795 ± 0.049 E6 | 2.800 ± 0.055 E5 | 2.318 ± 0.189 E5 | 3.705 ± 0.141 E7 | 5.894 ± 0.148 E6 | 1.805 ± 0.103 E5 |
| 5 | *E5* | 3.213 ± 0.123 E8 | 5.448 ± 0.205 E5 | 4.095 ± 1.821 E6 | 1.937 ± 0.517 E5 | 1.666 ± 0.064 E6 | 2.622 ± 0.034 E5 | 2.171 ± 0.154 E5 | 3.396 ± 0.279 E7 | 5.638 ± 0.192 E6 | 1.774 ± 0.100 E5 |
| 5 | *E6* | 3.078 ± 0.202 E8 | 5.252 ± 0.269 E5 | 4.911 ± 0.143 E6 | 1.807 ± 0.649 E5 | 1.607 ± 0.031 E6 | 2.501 ± 0.157 E5 | 1.912 ± 0.454 E5 | 3.311 ± 0.188 E7 | 5.273 ± 0.171 E6 | 1.621 ± 0.128 E5 |
| 5 | *E7* | 3.329 ± 0.117 E8 | 5.844 ± 0.132 E5 | 5.318 ± 0.047 E6 | 1.962 ± 0.924 E5 | 1.739 ± 0.083 E6 | 2.908 ± 0.034 E5 | 2.251 ± 0.213 E5 | 3.614 ± 0.120 E7 | 5.536 ± 0.038 E6 | 1.632 ± 0.031 E5 |
| 7 | *E1* | 1.871 ± 1.203 E6 | 6.256 ± 0.281 E4 | 2.143 ± 1.388 E4 | 2.132 ± 0.738 E3 | 1.646 ± 0.137 E5 | 1.349 ± 0.066 E5 | 0.000 ± 0.000 E0 | 2.072 ± 0.765 E5 | 3.598 ± 0.105 E6 | 1.415 ± 0.060 E5 |
| 7 | *E2* | 1.334 ± 0.089 E8 | 2.864 ± 1.232 E5 | 2.829 ± 0.568 E6 | 1.595 ± 0.434 E5 | 1.425 ± 0.188 E6 | 2.327 ± 0.484 E5 | 1.729 ± 0.357 E5 | 2.662 ± 0.662 E7 | 4.187 ± 0.671 E6 | 1.113 ± 0.201 E5 |
| 7 | *E3* | 3.180 ± 0.259 E8 | 5.338 ± 0.505 E5 | 5.433 ± 0.378 E6 | 1.795 ± 0.826 E5 | 1.537 ± 0.112 E6 | 2.395 ± 0.209 E5 | 1.588 ± 0.568 E5 | 3.064 ± 0.318 E7 | 5.408 ± 0.515 E6 | 1.757 ± 0.194 E5 |
| 8 | C | 3.002 ± 0.259 E8 | 4.524 ± 0.698 E5 | 4.802 ± 0.500 E6 | 1.696 ± 0.712 E5 | 1.503 ± 0.164 E6 | 2.393 ± 0.124 E5 | 1.916 ± 0.159 E5 | 3.094 ± 0.280 E7 | 4.954 ± 0.335 E6 | 1.467 ± 0.170 E5 |
| 8 | *E4* | 3.270 ± 0.116 E8 | 4.224 ± 2.066 E5 | 5.326 ± 0.068 E6 | 2.202 ± 0.330 E5 | 1.654 ± 0.053 E6 | 2.645 ± 0.032 E5 | 2.107 ± 0.086 E5 | 3.462 ± 0.141 E7 | 5.402 ± 0.113 E6 | 1.482 ± 0.046 E5 |
| 8 | *E5* | 3.261 ± 0.044 E8 | 4.867 ± 1.227 E5 | 5.454 ± 0.151 E6 | 1.886 ± 0.880 E5 | 1.790 ± 0.103 E6 | 2.590 ± 0.210 E5 | 1.967 ± 0.444 E5 | 3.390 ± 0.102 E7 | 5.489 ± 0.136 E6 | 1.570 ± 0.091 E5 |
| 9 | *E1* | 5.876 ± 3.705 E5 | 6.543 ± 0.396 E4 | 5.755 ± 1.134 E3 | 5.117 ± 1.740 E3 | 9.469 ± 0.077 E4 | 7.665 ± 0.455 E4 | 1.723 ± 2.985 E2 | 4.233 ± 1.746 E4 | 2.012 ± 0.072 E6 | 1.185 ± 0.073 E5 |
| 9 | *E2* | 8.152 ± 4.748 E6 | 4.119 ± 3.285 E4 | 4.824 ± 2.035 E5 | 3.863 ± 0.741 E4 | 1.422 ± 0.106 E6 | 2.144 ± 0.166 E5 | 1.127 ± 0.147 E5 | 1.946 ± 0.174 E7 | 4.275 ± 0.146 E6 | 1.215 ± 0.029 E5 |
| 9 | *E6* | 2.076 ± 0.209 E8 | 3.786 ± 0.346 E5 | 3.916 ± 0.384 E6 | 1.125 ± 0.558 E5 | 1.544 ± 0.129 E6 | 2.333 ± 0.190 E5 | 1.521 ± 0.093 E5 | 2.474 ± 0.268 E7 | 4.475 ± 0.551 E6 | 1.451 ± 0.131 E5 |
| 9 | *E7* | 3.145 ± 0.144 E8 | 4.800 ± 1.379 E5 | 3.334 ± 2.887 E6 | 1.217 ± 1.183 E5 | 1.736 ± 0.098 E6 | 2.685 ± 0.204 E5 | 2.215 ± 0.191 E5 | 3.522 ± 0.331 E7 | 5.167 ± 0.197 E6 | 1.270 ± 0.049 E5 |
| 10 | *E3* | 1.922 ± 0.539 E8 | 3.617 ± 0.348 E5 | 4.432 ± 0.179 E6 | 1.073 ± 0.374 E5 | 1.120 ± 0.081 E6 | 1.690 ± 0.165 E5 | 1.068 ± 0.276 E5 | 2.022 ± 0.244 E7 | 4.536 ± 0.097 E6 | 1.572 ± 0.153 E5 |
| 11 | *E4* | 2.339 ± 0.555 E8 | 4.422 ± 0.731 E5 | 4.389 ± 0.587 E6 | 1.819 ± 0.384 E5 | 1.712 ± 0.174 E6 | 2.441 ± 0.320 E5 | 1.854 ± 0.208 E5 | 2.724 ± 0.437 E7 | 4.592 ± 0.382 E6 | 1.121 ± 0.160 E5 |
| 11 | *E5* | 2.298 ± 0.803 E8 | 4.382 ± 0.756 E5 | 4.370 ± 0.616 E6 | 1.276 ± 0.639 E5 | 1.663 ± 0.145 E6 | 2.576 ± 0.064 E5 | 1.909 ± 0.210 E5 | 2.818 ± 0.100 E7 | 4.722 ± 0.080 E6 | 1.448 ± 0.127 E5 |
| 11 | *E6* | 3.026 ± 0.210 E7 | 7.446 ± 2.745 E4 | 1.188 ± 0.129 E6 | 3.483 ± 0.463 E4 | 1.214 ± 0.129 E6 | 1.970 ± 0.144 E5 | 7.088 ± 0.484 E4 | 9.202 ± 0.825 E6 | 2.428 ± 0.276 E6 | 1.363 ± 0.133 E5 |
| 12 | *E3* | 1.442 ± 0.306 E8 | 1.938 ± 0.845 E5 | 2.939 ± 0.958 E6 | 5.004 ± 1.240 E4 | 8.388 ± 1.481 E5 | 9.821 ± 3.090 E4 | 3.149 ± 3.064 E4 | 9.842 ± 3.764 E6 | 3.188 ± 0.619 E6 | 1.347 ± 0.057 E5 |
| 13 | *E2* | 6.910 ± 0.691 E5 | 3.349 ± 1.538 E3 | 4.301 ± 2.139 E3 | 1.979 ± 1.397 E3 | 7.949 ± 1.139 E5 | 1.211 ± 0.178 E5 | 5.168 ± 4.517 E2 | 2.087 ± 0.536 E6 | 2.818 ± 0.493 E6 | 1.152 ± 0.177 E5 |
| 13 | *E4* | 1.599 ± 0.242 E8 | 3.733 ± 0.999 E5 | 3.771 ± 0.907 E6 | 1.335 ± 0.723 E5 | 1.534 ± 0.044 E6 | 2.361 ± 0.147 E5 | 1.699 ± 0.226 E5 | 2.491 ± 0.494 E7 | 4.429 ± 0.076 E6 | 1.150 ± 0.033 E5 |
| 16 | C | 3.360 ± 0.135 E8 | 5.822 ± 0.272 E5 | 4.745 ± 1.474 E6 | 1.622 ± 0.732 E5 | 1.820 ± 0.028 E6 | 2.810 ± 0.126 E5 | 2.375 ± 0.137 E5 | 3.652 ± 0.265 E7 | 5.493 ± 0.065 E6 | 1.530 ± 0.106 E5 |
| 16 | *E1* | 6.485 ± 0.544 E5 | 5.256 ± 0.155 E4 | 4.717 ± 0.658 E3 | 3.220 ± 1.747 E3 | 6.344 ± 1.627 E4 | 2.120 ± 0.932 E4 | 0.000 ± 0.000 E0 | 8.242 ± 3.368 E3 | 9.391 ± 1.712 E5 | 5.907 ± 0.075 E4 |
| 16 | *E2* | 8.299 ± 1.357 E5 | 0.000 ± 0.000 E0 | 6.278 ± 1.733 E3 | 1.930 ± 1.253 E3 | 4.419 ± 0.325 E5 | 5.255 ± 0.312 E4 | 0.000 ± 0.000 E0 | 1.390 ± 0.367 E5 | 1.557 ± 0.086 E6 | 1.132 ± 0.098 E5 |
| 16 | *E3* | 1.146 ± 0.257 E7 | 4.415 ± 0.364 E4 | 5.039 ± 1.870 E5 | 3.247 ± 0.181 E3 | 1.281 ± 0.164 E5 | 4.417 ± 4.014 E2 | 0.000 ± 0.000 E0 | 1.732 ± 0.626 E5 | 3.569 ± 0.578 E5 | 7.792 ± 0.665 E4 |
| 16 | *E4* | 9.065 ± 0.506 E5 | 9.267 ± 7.785 E3 | 7.821 ± 2.294 E3 | 2.731 ± 1.556 E3 | 6.274 ± 2.109 E5 | 5.368 ± 2.946 E4 | 0.000 ± 0.000 E0 | 1.244 ± 0.906 E5 | 1.873 ± 0.649 E6 | 7.997 ± 2.412 E4 |
| 16 | *E5* | 5.148 ± 6.079 E7 | 1.404 ± 1.083 E5 | 1.596 ± 1.311 E6 | 2.849 ± 2.481 E4 | 1.469 ± 0.177 E6 | 2.328 ± 0.252 E5 | 9.716 ± 3.868 E4 | 1.733 ± 0.508 E7 | 3.957 ± 0.448 E6 | 1.332 ± 0.080 E5 |
| 16 | *E6* | 6.400 ± 2.949 E5 | 6.486 ± 0.387 E4 | 1.307 ± 0.549 E4 | 2.709 ± 1.728 E3 | 7.346 ± 1.059 E5 | 1.133 ± 0.212 E5 | 2.665 ± 4.616 E2 | 1.146 ± 0.493 E6 | 1.473 ± 0.252 E6 | 1.203 ± 0.121 E5 |
| 16 | *E7* | 3.562 ± 0.158 E8 | 6.028 ± 0.297 E5 | 5.789 ± 0.205 E6 | 2.199 ± 0.706 E5 | 1.789 ± 0.076 E6 | 3.170 ± 0.107 E5 | 2.454 ± 0.132 E5 | 3.707 ± 0.077 E7 | 5.116 ± 0.559 E6 | 8.057 ± 2.349 E4 |
