# Supplementary material for: Endophytic fungi from the roots of horseradish (Armoracia rusticana) and their interactions with the defensive metabolites of the glucosinolate - myrosinase - isothiocyanate system
Source: BMC Plant Biol. 2018 May 9;18:85. doi: 10.1186/s12870-018-1295-4 (PMC5944135; doi:10.1186/s12870-018-1295-4)
Supplement: Supplementary file 3 — Table S2. Residual amount of sinigrin and minor glucosinolates in horseradish extract after 16 days of incubation with endophytic fungi from horseradish and soil fungi. (DOC 33 kb) [file 12870_2018_1295_MOESM3_ESM.doc]

**Table S1.**

Residual amount of sinigrin and minor glucosinolates in horseradish extract after 16 days of incubation with endophytic fungi from horseradish and soil fungi. The residual amounts are expressed as percentage of control zero time (“C_0”). Treatments: C_0, zero time control; C_16, control (no fungal inoculation) after 16 days; ***E1-E7***: endophytes no. 1-7 (after 16 days); ***S1-S10***: soil fungi no. 1-10 (after 16 days). Glucosinolates side chain types: Aliphatic: Sinigrin, gluconapin, glucocochlearin, glucobrassicanapin; Methylthioalkyl: Glucoiberin, glucoibarin; aromatic: Glucotropaeolin, gluconasturtiin; indole: glucobrassicin, 4-Methoxyglucobrassicin.

| Treat-ment | Sinigrin | Gluconapin | Gluco-cochlearin | Gluco- brassicanapin | Glucoiberin | Glucoibarin | Gluco- tropaeolin | Gluco- nasturtiin | Gluco-brassicin | 4-Methoxy- glucobrassicin |
| --- | --- | --- | --- | --- | --- | --- | --- | --- | --- | --- |
| C_0 | 100 ± 1.6% | 100 ± 3.6% | 100 ± 5.9% | 100 ± 63.4% | 100 ± 4.3% | 100 ± 3.3% | 100 ± 6% | 100 ± 2.3% | 100 ± 3.7% | 100 ± 3.6% |
| C_16 | 104.7 ± 4.2% | 104.1 ± 4.9% | 87.6 ± 35% | 115.7 ± 52.2% | 105.4 ± 1.6% | 105.8 ± 4.7% | 109.9 ± 6.3% | 106.5 ± 7.7% | 99.3 ± 1.2% | 83.1 ± 5.8% |
| ***E1*** | 0.2 ± 0% | 9.5 ± 0.1% | 0.1 ± 0% | 2.5 ± 0.9% | 3.7 ± 0.9% | 8.1 ± 3.5% | 0 ± 0% | 0 ± 0% | 17 ± 3.1% | 32.1 ± 0.4% |
| ***E2*** | 0.3 ± 0% | 0 ± 0% | 0.1 ± 0% | 1.7 ± 0.6% | 25.6 ± 1.9% | 19.8 ± 1.2% | 0 ± 0% | 0.4 ± 0.1% | 28.1 ± 1.6% | 61.5 ± 5.3% |
| ***E3*** | 3.6 ± 0.8% | 8.2 ± 1% | 8.9 ± 4.9% | 1.8 ± 0.4% | 7.4 ± 0.9% | 0.2 ± 0.2% | 0.1 ± 0.2% | 0.5 ± 0.2% | 6.5 ± 1% | 42.3 ± 3.6% |
| ***E4*** | 0.3 ± 0% | 1.8 ± 1.5% | 0.1 ± 0% | 1.4 ± 1% | 36.3 ± 12.2% | 20.3 ± 11.1% | 0 ± 0% | 0.4 ± 0.3% | 33.9 ± 11.7% | 43.4 ± 13.1% |
| ***E5*** | 16 ± 18.9% | 25.2 ± 19.2% | 30.8 ± 25.3% | 20.1 ± 17.5% | 85 ± 10.3% | 87.6 ± 9.5% | 45 ± 17.9% | 50.5 ± 14.8% | 71.6 ± 8.1% | 72.3 ± 4.3% |
| ***E6*** | 0.2 ± 0.1% | 11.8 ± 0.9% | 0.3 ± 0.1% | 2.1 ± 0.5% | 42.5 ± 6.1% | 42.6 ± 8% | 0.1 ± 0.2% | 3.3 ± 1.4% | 26.6 ± 4.6% | 65.3 ± 6.6% |
| ***E7*** | 110.9 ± 4.9% | 107.7 ± 5.3% | 111.6 ± 3.9% | 149.4 ± 63% | 103.5 ± 4.4% | 119.3 ± 4% | 113.6 ± 6.1% | 108.1 ± 2.3% | 92.5 ± 10.1% | 43.7 ± 12.8% |
| ***S1*** | 0.1 ± 0% | 0.3 ± 0.1% | 0.1 ± 0% | 0.9 ± 0.3% | 5.1 ± 0.2% | 32 ± 0.9% | 0.6 ± 0.4% | 0 ± 0% | 65.3 ± 3.7% | 79.3 ± 3.1% |
| ***S2*** | 0.1 ± 0% | 0 ± 0% | 0.1 ± 0% | 0.8 ± 0.2% | 0 ± 0% | 0 ± 0% | 0.4 ± 0.1% | 0 ± 0% | 0 ± 0% | 0.4 ± 0.1% |
| ***S3*** | 85.2 ± 4.9% | 78.7 ± 1.3% | 88.3 ± 2.9% | 70 ± 23.8% | 75 ± 1.9% | 72.7 ± 3% | 55.3 ± 2.3% | 74.8 ± 0.6% | 83.9 ± 1% | 88 ± 4.4% |
| ***S4*** | 58.7 ± 5.7% | 52.4 ± 3.8% | 56.9 ± 4.4% | 55.1 ± 26.6% | 72 ± 5% | 84.6 ± 4.1% | 61.8 ± 3.7% | 33.4 ± 5.7% | 47.3 ± 4.7% | 81.4 ± 2.3% |
| ***S5*** | 87.5 ± 18.2% | 96.4 ± 10.4% | 101.2 ± 7.5% | 111.8 ± 19.2% | 101.4 ± 4.6% | 102.5 ± 3.8% | 104.2 ± 9.5% | 106.7 ± 3.8% | 98.7 ± 2% | 92.8 ± 4.4% |
| ***S6*** | 67.3 ± 12.1% | 59.3 ± 18.1% | 72.5 ± 14% | 70.8 ± 23.9% | 61.8 ± 9.4% | 65.6 ± 13.9% | 73.8 ± 12% | 67.4 ± 14.4% | 63.2 ± 10.4% | 40.3 ± 9.6% |
| ***S7*** | 105.2 ± 4.9% | 95.8 ± 3.9% | 91.7 ± 15% | 118.2 ± 6.8% | 90.3 ± 3.4% | 92.7 ± 2.8% | 97.3 ± 8.2% | 95.7 ± 3.6% | 89.8 ± 2.9% | 79.2 ± 2.9% |
| ***S8*** | 100.8 ± 2.5% | 91.4 ± 4.4% | 95.5 ± 5% | 110.9 ± 6.9% | 88 ± 4.8% | 92.2 ± 4.8% | 92.8 ± 7.5% | 87.9 ± 4% | 92.2 ± 3.5% | 84.3 ± 6.2% |
| ***S9*** | 0.3 ± 0.2% | 0.2 ± 0.1% | 0.2 ± 0.2% | 0.7 ± 0.2% | 0.1 ± 0.2% | 0 ± 0% | 0.5 ± 0.3% | 0.2 ± 0.1% | 0.4 ± 0.1% | 32.6 ± 1.3% |
| ***S10*** | 0.1 ± 0.1% | 0.1 ± 0.1% | 0.1 ± 0% | 1 ± 0.3% | 0.1 ± 0.1% | 0 ± 0% | 0.4 ± 0.3% | 0.1 ± 0% | 0.2 ± 0% | 39.9 ± 11.2% |
